# The ornithine-urea cycle involves fumaric acid biosynthesis in *Aureobasidium pullulans* var. *aubasidani*, a green and eco-friendly process for fumaric acid production

**DOI:** 10.1016/j.synbio.2022.10.004

**Published:** 2022-10-19

**Authors:** Xin Wei, Miao Zhang, Guang-Yuan Wang, Guang-Lei Liu, Zhen-Ming Chi, Zhe Chi

**Affiliations:** a College of Marine Life Sciences, Ocean University of China, Yushan Road, No. 5, Qingdao, China; b Laboratory for Marine Biology and Biotechnology, Qingdao National Laboratory for Marine Science and Technology, 266003, China; c College of Life Science, Shandong Province Key Laboratory of Applied Mycology, Qingdao Agricultural University, Qingdao, 266109, China

**Keywords:** Fumaric acid, *A. pullulans* var. *aubasidani*, Ornithine-urea cycle, Ca^2+^signaling pathway, FA, Fumaric acid, OUC, ornithine-urea cycle, Cps, carbamoyl phosphate synthase, Otc, ornithine transcarbamoylase, Ass, argininosuccinate synthase, Ast, argininosuccinate lyase, Arg, arginase, AG, N-acetyl glutamate

## Abstract

The current petroleum chemical methods for fumaric acid production can cause heavy pollution and global warming. In this study, the engineered strains of *A. pullulans* var. *aubasidani* were found to be suitable for green fumaric acid producer. Removal and complementation of the relevant genes showed only the ornithine-urea cycle (OUC) was involved in high level fumarate biosynthesis which was controlled by the Ca^2+^ signaling pathway. Removal of both the *GOX* gene encoding glucose oxidase and the *PKS1* gene encoding the polyketide synthase for 3,5-dihydroxydecanoic acid biosynthesis and overexpression of the *PYC* gene encoding pyruvate carboxylase made the strain e-PYC produce 88.1 ± 4.3 g/L of fumarate at flask level and 93.9 ± 0.8 g/L of fumarate during the fed-batch fermentation. As a yeast-like fungal strain, it was very easy to cultivate *A. pullulans* var. *aubasidani* DH177 and their mutants in the bioreactor and to edit its genomic DNAs to enhance fumarate production. It was found that 2 mol of CO_2_ could be fixed during a maximal theoretical yield of 2 mol of fumarate per mole of glucose consumed in the OUC. Therefore, the OUC-mediated fumarate biosynthesis pathway in *A. pullulans* var. *aubasidani* was a green and eco-friendly process for the global sustainable development and carbon neutrality.

## 1 Introduction

It has been well known that fumaric acid, (E)-2-butenedioic acid with a carbon-carbon double bond and two carboxylic acid groups, has many applications. Fumarate can be used as a food additive and acidulant, as a main component of paper resins, unsaturated polyester resins and plasticizers, as a precursor for fumaric acid esters synthesis for treatment of psoriasis, sclerosis and human cancer [2], as a support material for tissue engineering. Currently, fumarate is solely synthesized by petrochemical methods via hydrolysis of maleic anhydride which is derived by the oxidation of butane or benzene in the presence of vanadyl pyrophosphate as a catalyst [28]. However, this chemical process has many drawbacks, such as high temperature reaction, the formation of toxic carbon monoxide and release of greenhouse gas carbon dioxide, which contribute to environmental pollution and global warming. Because of the very high price and continuous depletion of crude oil in the earth and the recent attention to green chemistry for sustainable development and carbon neutrality, an alternative process which ensures sustainable development by reducing reliance on fossil fuels burning from which CO_2_ emissions are the leading causes of global warming and climate change and increasing bio-production is urgently required. Therefore, many researchers and commercial manufacturers have had great interests in bio-production of fumarate, a sustainable and eco-friendly alternative to the petroleum-based production [29].

**Fig. 1:**
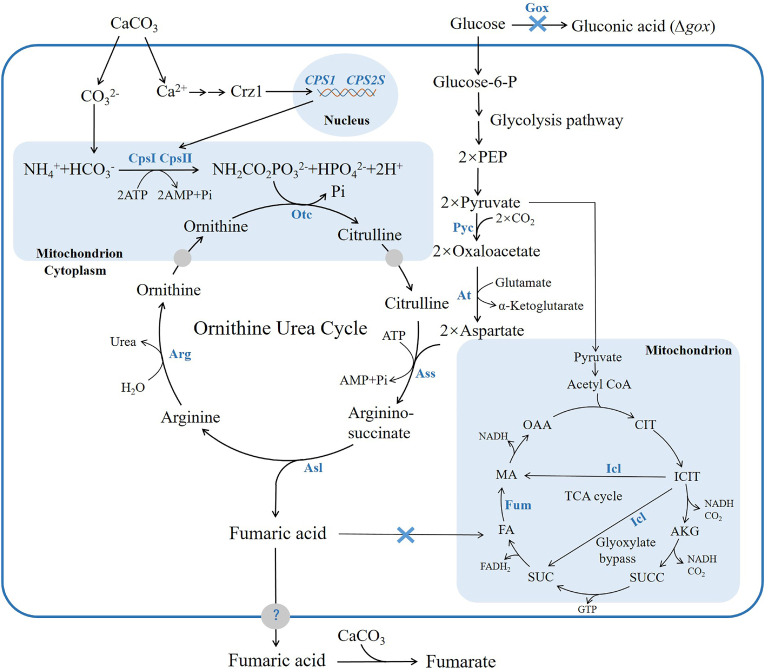
Fumaric acid biosynthesis from the OUC and TCA cycle Pyc: Pyruvate carboxylase; At: Aspatrate aminotransferase; Cps: Carbamoyl phosphate synthase; Otc: Ornithine transcarbamoylase; Ass: Argininosuccinate synthase; Asl: Argininosuccinate lyase; Arg: Arginase; Sfc: Succinate-fumarate carrier; Icl: Isocitrate lyase: Fum: Fumarase.

It has been well confirmed that *Rhizopus oryzae* is being the dominant microbial fumarate overproducer [39]. However, the fungal strain has been found to have many disadvantages, such as low fungal growth, low yield of fumarate, clumps and pellets formation, simultaneous production of ethanol and lactate, the complexities of this fungal strain mediated fumaric acid production and difficulties in genetic modification, cell growth control, the control of the pellet size and oxygen transfer [28]. All these have been a major obstacle for the commercialization of fumarate bio-production by *R. oryzae* [21]. These have led researchers to find the possibility of using alternate microorganisms for the bio-production of fumarate, particularly using *Escherichia coli* and *Saccharomyces cerevisiae* to produce fumarate [28]. Unfortunately, the fumarate titers obtained using genetically engineered strains of *E. coli*, *S. cerevisiae*, *T. glabrata* and *Scheffersomyces stipitis* are significantly lower than that produced by the wild type strain *R. oryzae* [14]. Recently, it has been reported that the yeast-like fungi *Aureobasidium pullulans* var. *aubasidani* DH177 isolated from the leaves of *Weigela florida* in China accumulated 64.7% (w/w) oil in its cells, 22.4 g/L cell biomass and 32.3 g/L fumarate during a 5-L batch fermentation [34]. In our previous studies [45,46], it has been well confirmed that the whole genomes of different strains of *Aureobasidium* spp. can be easily and successfully edited using the efficient Cre/loxp site-specific recombination system constructed in this laboratory. It has been also shown that there are many potential advantages of *Aureobasidium* spp. for developing the cell factory for fungal biotechnology and biology over *R. oryzae*, *S. cerevisiae* and any other fungi [10,35]. In the present study, the genetic modification of *A. pullulans* var. *aubasidani* DH177, the new fumarate producer, could render the engineered strain to produce over 93.0 g/L of fumarate. Especially, it was confirmed that the ornithine-urea cycle (OUC) was involved in high level fumarate biosynthesis in *A. pullulans* var. *aubasidani* under the control of Ca^2+^ signaling pathway. Therefore, 2 mol of CO_2_ could be fixed theoretically during a maximal yield of 2 mol of fumarate per mole of glucose consumed (Fig. 1). So, this would give us a new hope that the engineered strains of *A. pullulans* var. *aubasidani* DH177, much better fumarate overproducer than *R. oryzae,* could be used for feasible fumarate bio-production in a large scale in the future and the new green process for fumarate bio-production would be obtained for green sustainable development and carbon neutrality.

In recent years, the ornithine-urea cycle (OUC) has received great interest because it has many physiological functions in all the organisms [1,24,43]. All the reactions involved in the OUC in yeasts are shown in Fig. 1. From Fig. 1, it can be seen that six enzymes, CpsI: carbamoyl phosphate synthase I; CpsII: carbamoyl phosphate synthase II; Otc: ornithine transcarbamoylase; Ass: argininosuccinate synthase; Asl: argininosuccinate lyase; Arg: arginase, are responsible for these reactions. In addition, N-acetyl glutamate (AG) acts as an allosteric effector that influences the monomer-dimer association-dissociation of CpsI. Under normal conditions of citrulline biosynthesis, CpsI activity is rate-limiting and dependent on AG concentration. Moreover, the activity of CpsI system can be affected by concentrations of ATP, ammonia, bicarbonate and Mg^2+^ in mitochondrion. In turn, the requirements of acetyl-CoA and glutamate for the synthesis of AG makes it dependent on oxidation of pyruvate and fatty acids for generation of acetyl-CoA, and glutamate dehydrogenase and transaminases for maintaining glutamate levels.

## 2 Materials and methods

### 2.1 The microbial strains, plasmids and media

The yeast-like fungal strain *A. pullulans* var. *aubasidani* DH177 was obtained from the leaves of *Weigela florida* in China [34]. The competent cells of *E. coli* DH5α were prepared in this laboratory. The plasmid pMD19-T for amplifying plasmids in *E. coli* was purchased from TaKaRa Company in Dalian, China. The plasmid pLB-simple was bought from the TIANGEN company in Beijing, China. The disruption vector pFL4A-NAT-loxp carrying the nourseothricin resistance gene (*NAT* gene), the expression vector pNATX13-NS carrying the *NAT* gene and the plasmid pAMCRE-1 carrying the autonomously replicating DNA sequence, Cre recombinase gene and hygromycin B resistance gene (*HPT* gene) were constructed in this laboratory [9,22,45]. The plasmid pAPX13-gfp carrying the *GFP* gene was kindly offered by Professor Long-Fei Wu from CNRS, France. The YPD medium for cultivation of the yeast-like fungal strains contained 10.0 g/L yeast extract, 20.0 g/L peptone, 20.0 g/L glucose. The YPD medium with 1.0 M sorbitol was used to cultivate the transformants. The fumarate production medium contained 120.0 g/L glucose, 2.0 g/L, (NH_4_)_2_SO_4_, 0.3 g/L, KH_2_PO_4_, 0.3 g/L MgSO_4_•7H_2_O, 0.3 g/L ZnSO_4_, 80.0 g/L CaCO_3._ LB medium for cultivation of *E. coli* contained 5.0 g/L yeast extract, 10.0 g/L NaCl, 10.0 g/L tryptone. LA medium was the LB medium with 100.0 μg/mL of ampicillin. The medium for cultivation of the transformants of the yeast-like fungal strain was the YPD medium with 100.0 μg/mL of nourseothricin or hygromycin B.

### 2.2 Isolation and sequencing of the genomic DNAs and molecular identification of the DH177 strain

The genomic DNAs of *A. pullulans* var. *aubasidani* DH177 were isolated and purified based on the methods described by Ref. [9]. The purified DNAs were detected by the agarose gel electrophoresis and quantified using a Qubit® 2.0 Fluorometer (Thermo Scientific). The libraries for single-molecule real-time (SMRT) sequencing were constructed with an insert size of 20 kb using a SMRT bell TM Template kit (version 1.0). For the Illumina HiSeq sequencing, sequencing libraries were generated using a NEBNext® Ultra™ DNA Library Prep Kit for Illumina (NEB, USA) following manufacturer's recommendations and index codes were added to attribute sequences to each sample. The whole genomic DNAs of the DH177 strain were sequenced using a PacBio Sequel platform and Illumina NovaSeq PE150 at the Beijing Novogene Bioinformatics Technology Co., Ltd, China. In order to ensure the accuracy of the subsequent analysis results, the low-quality reads were filtered (≤500 bp) to obtain clean data. The errors in the primary assembly were identified and corrected with a BLASR v5.1 [3]. The whole-genome based phylogenetic tree of *Aureobasidium* spp. including *A. pullulans* var. *aubasidani* CBS 100524 [32] was conducted through a composition vector (CV) approach on the CVTree3 website (http://tlife.fudan.edu.cn/cvtree/cvtree/).

### 2.3 Cloning and characterization of the relevant genes

The *GOX*, *FAA*, *ADSL*, *FUM*, *SFC*, *ICL1*, *ICL2*, *ASL*, *CPS1, CPS2L, CPS2S, CRZ1*, *PKS1* and *PYC1* gene (Table S1) were PCR amplified using the primers shown in Table S2 and the genomic DNAs or cDNAs of the DH177 strain as templates. The conserved DNA and amino acid sequences of the cloned genes were analyzed and characterized using the software in Table S3.

### 2.4 Construction of disruption and expression vectors

It was found that only in the presence of CaCO_3_, *A. pullulans* var. *aubasidani* DH177 could produce a large amount of calcium gluconate (Ca^2+^-GA). In order to block gluconate production, pFL4A-NAT-loxp-*ΔGOX* (Table S1 and Fig. S1A) was constructed to delete the key *GOX* gene encoding glucose oxidase [46] (Fig. 1). It has been reported that fumaric acid in fungi could be synthesized through six pathways: TCA cycle, glyoxylate cycle, cytoplasmic reductive pathway, TTP metabolism, purine metabolism and OUC [14]. In order to explore whether the OUC was involved in fumaric acid biosynthesis, pFL4A-NAT-loxp-*ΔASL* (Table S1 and Fig. S4H)*,* pFL4A-NAT-loxp-*ΔCPS1* (Table S1 and Fig. S1I), pFL4A-NAT-loxp-*ΔCPS2L* (Table S1 and Fig. S1J) and pFL4A-NAT-loxp-*ΔCPS2S* (Table S1 and Fig. S1K) were constructed for removal of the key *ASL*, *CPS1, CPS2L* and *CPS2S* genes in the OUC (Fig. 1). In order to explore whether TTP metabolism was involved in fumaric acid synthesis, pFL4A-NAT-loxp-*ΔFAA* (Table S1 and Fig. S1B) was constructed to abolish the key *FAA* gene. To reveal whether purine metabolism was implicated with fumaric acid synthesis, pFL4A-NAT-loxp-*ΔADSL* (Table S1 and Fig. S1C) was constructed to delete the key *ADSL* gene*.* To confirm whether the TCA cycle was associated with fumaric acid biosynthesis, pFL4A-NAT-loxp-*ΔFUM* (Table S1 and Fig. S1D) and pFL4A-NAT-loxp-*ΔSFC* (Table S1 and Fig. S1E) were constructed to delete the *FUM* gene and the *SFC* gene. In order to get the evidence to show whether the glyoxylate cycle took part in fumaric acid biosynthesis, pFL4A-NAT-loxp-*ΔICL1* (Table S1 and Fig. S1F) and pFL4A-NAT-loxp-*ΔICL2* (Table S1 and Fig. S1G) were constructed to clean the two key genes *ICL1* and *ICL2.* It was found that the presence of CaCO_3_ is required for fumaric acid production [34]. In order to demonstrate whether the Ca^2+^ signaling pathway regulates fumaric acid biosynthesis [33], pFL4A-NAT-loxp-*△CRZ1* (Fig. S1L) was constructed to delete the key *CRZ1* gene for transcriptional activator Crz1*.* To show whether acetyl-CoA for fatty acid biosynthesis was implicated with fumaric acid biosynthesis, pFL4A-NAT-loxp-*ΔPKS1* (Fig. S1M) was constructed to abolish the *PKS1* gene. All the corresponding primers and restriction enzymes are shown in Table S2 and Fig. S1. The functions of all the genes used in this study were indicated in Table S1.

In order to express the *ASL*, *CPS1*, *CPS2L*, *CPS2S*, *CRZ1-GFP*, *SFC* and *PYC* genes in the relevant strains, they were PCR amplified using the primers ASL-F/ASL-R, CPS1–F/CPS1-R, CPS2L-F/CPS2L-R, CPS2S–F/CPS2S-R, CRZ1-F/CRZ1-R, GFP-F/GFP-R, SFC-F/SFC-R and PYC-F/PYC-R, respectively (Table S2). The PCR products were hydrolyzed with the enzymes *Mlu*I/*Pst*I, *Spe*I/*Mlu*I, *Afl*II/*Sac*I, *Sac*I/*Sal*I, *Sac*I/*Sal*I, *Sac*I/S*al*I, *Spe*I/*Afl*II and *Mlu*I/*Pst*I, respectively. The hydrolyzed PCR products were linked into pNATX13-NS hydrolyzed with the same enzymes, forming pNATX13-NS-*ASL* (Fig. S1N)*,* pNATX13-NS-*CPS1* (Fig. S1O)*,* pNATX13-NS-*CPS2L* (Fig. S1S), pNATX13-NS-*CPS2S* (Fig. S1T), pNATX13-NS-*CRZ1-GFP* (Fig. S1P)*,* pNATX13-NS-*SFC* (Fig. S1Q) and pNATX13-NS-*PYC1* (Fig. S1R).

### 2.5 Transformation and isolation of disruptants and expressing strains

The linear DNA fragments 5′-arm-loxp-polyA-NAT-PGK-loxp-3′-arm were PCR amplified from the pFL4A-NAT-loxp-*ΔGOX*, pFL4A-NAT-loxp-*ΔFAA,* pFL4A-NAT-loxp-*ΔADSL,* pFL4A-NAT-loxp-*ΔFUM,* pFL4A-NAT-loxp-*ΔSFC*, pFL4A-NAT-loxp-*ΔICL1*, pFL4A-NAT-loxp-*ΔICL2,* pFL4A-NAT-loxp-*ΔASL*, pFL4A-NAT-loxp-*ΔCPS1*, pFL4A-NAT-loxp-*ΔCPS2L,* pFL4A-NAT-loxp-*ΔCPS2S*, pFL4A-NAT-loxp-*ΔCRZ1* and pFL4A-NAT-loxp-*ΔPKS1* obtained above using the primers GOX-5F/GOX-3R, FUMARATEA-5F/FUMARATEA-3R, ADSL-5F/ADSL-3R, FUM-5F/FUM-3R, SFC-5F/SFC-3R, ICL1-5F/ICL1-3R, ICL2-5F/ICL2-3R, ASL-5F/ASL-3R, CPS1–5F/CPS1-3R, CPS2L-5F/CPS2L-3R, CPS2S–5F/CPS2S-3R, CRZ1-5F/CRZ1-3R and PKS1–5F/PKS1-3R (Table S2), respectively. The obtained linear DNA fragment 5′-arm-loxp-polyA-NAT-PGK-loxp-3′-arm from the pFL4A-NAT-loxp-*△GOX* was transformed into the competent cells of the wild type strain *A. pullulans* var. *aubasidani* DH177 and the *Δgox* mutant was obtained. Then, the pAMCRE-1 plasmid was transformed into the *Δgox* mutant strain to remove the *NAT* and *HPT* genes (Table S4). The linear DNA fragments 5′-arm-loxp-polyA-NAT-PGK-loxp-3′-arm from the pFL4A-NAT-loxp-*△PKS1* were introduced into the competent cells of the *Δgox* mutant and the *ΔgoxΔpks1* mutant was obtained. Similarly, the *NAT* and *HPT* genes were removed by using the pAMCRE-1 plasmid (Table S4). In the same principle, the linear DNA fragments 5′-arm-loxp-polyA-NAT-PGK-loxp-3′-arm from the pFL4A-NAT-loxp-*△FAA*, the pFL4A-NAT-loxp-*△ADSL*, the pFL4A-NAT-loxp-*△FUM*, the pFL4A-NAT-loxp-*△SFC*, the pFL4A-NAT-loxp-*△ICL1*, the pFL4A-NAT-loxp-*△ICL2,* the pFL4A-NAT-loxp-*△ASL*, the pFL4A-NAT-loxp-*△CPS1,* pFL4A-NAT-loxp-*△CPS2L*, pFL4A-NAT-loxp-*△CPS2S* and the pFL4A-NAT-loxp-*△CRZ1* were introduced into the competent cells of the *Δgox* mutant, respectively and different disruptants (Δgox*Δfaa*, Δgox*Δadsl,* Δgox*Δfum,* Δgox*Δsfc,* Δgox*Δicl1,* Δgox*Δicl2,* Δgox*Δasl,* Δgox*Δcps1,* Δgox*Δcps2l,* Δgox*Δcps2s* and *ΔgoxΔcrz1*) were obtained (Table S4), respectively. Then, the pAMCRE-1 plasmid was transformed into these disruptants to remove the *NAT* and *HPT* genes (Table S4).

After the plasmids pNATX13-NS-*ASL*,pNATX13-NS-*CPS1*, pNATX13-NS-*CRZ1-GFP,* pNATX13-NS-*SFC*,pNATX13-NS-*PYC*,pNATX13-NS-*CPS2L* and pNATX13-NS-*CPS2S* were digested with the enzyme *Sma*Ⅰ, the linear 18SrDNA-TEF-ASL-loxp-polyA-NAT-PGK-loxp-26SrDNA,18SrDNA-TEF-CPS1-loxp-polyA-NAT-PGK-loxp-26SrDNA,18SrDNA-TEF-CRZ1-GFP-loxp-polyA-NAT-PGK-loxp-26SrDNA,18SrDNA-TEF–SFC–loxp-polyA-NAT-PGK-loxp-26SrDNA, 18SrDNA-TEF-PYC-loxp-polyA-NAT-PGK-loxp-26SrDNA, 18SrDNA-TEF-CPS2L-loxp-polyA-NAT-PGK-loxp-26SrDNA and 18SrDNA-TEF-CPS2S-loxp-polyA-NAT-PGK-loxp-26SrDNA obtained were transformed into the competent cells of *ΔgoxΔasl*, *ΔgoxΔcps1*, *ΔgoxΔcrz1, ΔgoxΔsfc, ΔgoxΔpks1, ΔgoxΔcps2l* and *ΔgoxΔcps2s* without *NAT* gene*,* respectively, resulting in the transformants named ASL-H, CPS1–H, CRZ1-H, SFC-H, e-PYC, CPS2L-H and CPS2S–H (Table S4).

### 2.6 Calcium fumarate production and identification of the produced fumaric acid

*A. pullulans* var. *aubasidani* DH177, all the disruptants and transformants (Table S4) obtained above were aerobically cultivated in 50.0 mL of the liquid YPD medium at 28 °C and 180 rpm for 24 h. The seed culture (5.0 mL) was transferred to 45.0 mL of the fumarate production medium and the new culture was continued to be grown at 28 °C and 180 rpm for 7 days, respectively. Each culture (10.0 mL) was centrifuged at 5000×*g* for 20 min. All the fumarate in the supernatant was precipitated using cold methanol and dissolved in distilled water based on the methods described by Ref. [34] and the same procedures were repeated three times. The purified fumarate was dried and weighed and the amount of fumarate per liter of culture was calculated. At the same time, cell dry weight in the culture was assayed and calculated according to the methods described by Ref. [8]. The fumarate produced by the *Δgox* mutant (Table S4) was further purified. The calcium in the purified fumarate was totally removed by addition of 0.1 N sulfuric acid and the formed calcium sulfate was eliminated by centrifugation at 10,000×*g* for 10 min and filtration using 0.22 μm membrane. The supernatant obtained was incubated at 4 °C and fumaric acid was crystalized. The crystals were dissolved in pure water at 80 °C and the solution was incubated at 4 °C and fumaric acid was crystalized again. The procedures were repeated several times until fumaric acid was pure [4]. The purified fumaric acid was esterized based on the methods described by Ref. [6]. Fifty mg of the fumaric acid crystal was mixed with 2.5 mL of 2.0% (v/v) H_2_SO_4_ -methanol solution in the sealed bottle. The mixture was heated at 80 °C with mild mixing for 1.5 h. Then, the mixture was cooled to room temperature. The fumaric acid methyl esters formed were extracted with n-hexane and the extracts were filtered using 0.22 μm membrane, the filtrate was analyzed using a GC-MS and their molecular weight was obtained. The injected sample was 1.00 μL into an Agilent 7890A/5975C machine, the column was Agilent HP-INNOWax Polyethylene Glyco (30 m × 50 μm × 0.25 μm), the initial temperature was 100 °C and then was increased to 240 °C at the rate of 15 °C/min within 20 min, and the running time was 20 min.

### 2.7 Determination of transcriptional levels of relevant genes in the wild type strain, the disruptant and overexpressing strains

*A. pullulans* var. *aubasidani* DH177 and its various deletants and transformants obtained above (Table S4) were cultivated in 50.0 mL of the liquid YPD medium at 28 °C and 180 rpm for 24 h. The seed culture (5.0 mL) was transferred to 45.0 mL of the fumarate production medium and the new culture was continued to be grown at 28 °C and 180 rpm for 3 d. The yeast-like fungal cells in the cultures were harvested and washed with sterile distilled water by centrifugation at 8000×*g* for 5 min. The total RNAs in the washed cells were extracted using a Fungal RNA prep pure kit (OMEGA, USA) and the bands of the RNAs were checked by agarose electrophoresis. Reverse transcription was performed using a PrimeScript RT reagent Kit (TaKaRa, Japan) according to the manufacturer's protocol. The fluorescent real-time RT-PCR assay was carried out using a Rotor-Gene Q RT-PCR analyzer (QIAGEN Hilden, Germany) in triplicate based on the methods described by Ref. [20]. All the primers for the fluorescent real-time PCR are shown in Table S5. The relative expression quantity was calculated using the formula RATE = 2^-ΔΔCt^ and Rotor-Gene Q 2.0.2 Real-time Date Acquisition and Analysis Software. The sample data obtained from the real-time PCR analysis were subjected to One-way Analysis of Variance (ANOVA) [15]. P values were calculated by Student's t-test (n = 3). P values less than 0.05 were considered statistically significant. Statistical analysis was performed using a SPSS 11.5 for Windows (SPSS Inc., Chicago, IL).

### 2.8 Intracellular Crz1-Gfp localization in response to CaCO_3_

The *GFP* gene with the linker GGSGGGSG was PCR amplified from the plasmid pAPX13-gfp carrying the *GFP* gene as the template using the primers GFP-F/GFP-R. The *CRZ1* gene without the termination codon was amplified from the cDNA of the wild type strain DH177 as the template using the primers CRZ1-F/CRZ1-R (Table S2), respectively. The two PCR products (the *GFP* gene and the *CRZ1* gene) were digested with the enzymes *Afl*Ⅱ/*Sac*I and *Sac*I*/Sal*I and the digested PCR products were ligated into the plasmid pNATX13-NS digested with the same enzymes, yielding the recombinant plasmids pNATX13-NS-*CRZ1*-*GFP* (Fig. S1P)*.* The recombinant plasmids were digested with the corresponding DNA restriction enzyme *Sma*I, and the linear fragments obtained were transformed into the competent cells of the transformant *ΔgoxΔcrz1* obtained above. The new transformants obtained were grown in the fumarate production medium with CaCO_3_ and without CaCO_3_ for 24 h, respectively and the amounts of the produced fumarate and cell dry weight were measured as described above. The yeast-like fungal cell nuclei were stained immediately by the addition of 20.0 μL DAPI (4,6-diamidino-2-phenylindole) solution (30.0 μg/mL) to 200 μL of the cultures, and the cultures were incubated at 28 °C for 20 min before microscopy. Finally, the treated cells were observed under blue light with a Leica confocal microscope with the × 100 oil immersion objective. Images were recorded using the CellSense Standard software. The stained cell nuclei (blue ones) were visualized at an excitation wavelength of 405 nm, whereas the Gfp proteins (green ones) in the cells were observed at an excitation wavelength of 488 nm [16].

### 2.9 10-Liter fed-batch fermentation using the e-PYC strain

The production medium and cultivation conditions for the e-PYC strain obtained above were optimized to produce the maximum fumarate by the orthogonal test. In the orthogonal test, six factors and three levels were designed to optimize the concentrations of glucose, ammonium sulfate, calcium carbonate, potassium dihydrogen phosphate, magnesium sulfate heptahydrate and zinc sulfate in the fumarate production medium. 6.0% (v/v), 8.0% (v/v), 10.0% (v/v), 12.0% (v/v) and 14.0% (v/v) of the e-PYC strain seed culture were inoculated into the optimized production medium, respectively. Finally, the e-PYC strain was cultivated in 50.0 mL of the optimized fumarate production medium of the 250-mL flask at 28 °C and 180 rpm for 168 h. During the cultivation, the fumarate titer, cell dry weight and residual glucose concentration of the culture were measured every 24 h as described above and below.

A 10-Litter fermenter (bioq-6005-6010b, Shanghai huihetang Bioengineering Equipment Co., Ltd. Shanghai, China) was used for the fermentation. The e-PYC strain was cultured in the YPD liquid medium at 28 °C and 180 rpm for 24 h. Then, 700.0 mL of the seed culture (the cell concentration 1.0 × 10^8^ cells/mL) was inoculated into 6.3 L of the optimized fumarate production medium of the 10-L fermenter and cultivated at temperature of 28 °C, agitation of 280 rpm, aeration of 300 L/h for 168 h. During the fed batch fermentation, additional 200 g of sterile glucose was added into the fermenter at 120 h. 40.0 mL of the culture was sampled every 12 h to determine the cell dry weight and the amount of fumarate as described above and the residual glucose concentration in the culture was quantitatively measured based on the methods described by Ref. [30].

## 3 Results

### 3.1 Sequencing of the genomic DNA of the DH177 strain and its molecular identification

**Fig. 2:**
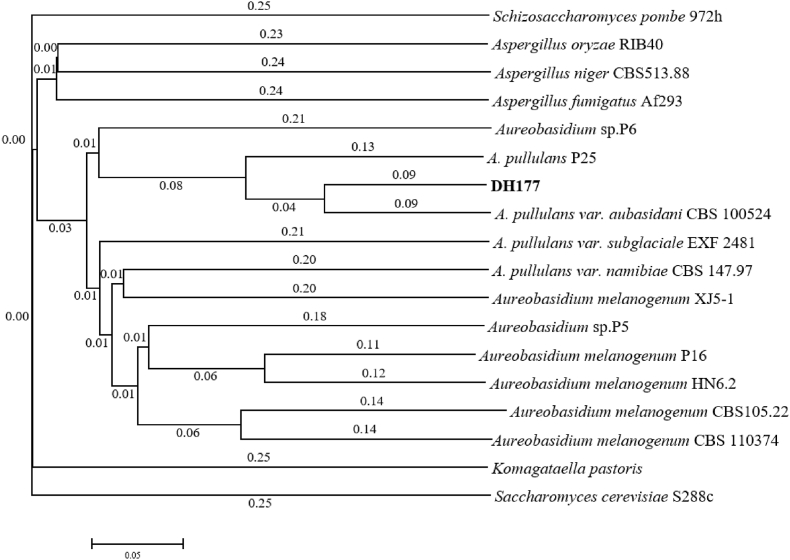
The phylogenetical tree of the whole-genomic DNA sequences from the DH177 strain, *A. pullulans* var. *aubasidani* CBS 100524, *A. pullulans* P25 and any other *Aureobasidium* spp. and other fungal strains.

The genomic DNA sequence of the DH177 strain showed that the size of its genome was 31.34 Mbp, the whole genome contained 144 scaffolds and the GC content of the genome was 50.3% (data not shown). The whole genome sequence of the DH177 strain was deposited at the NCBI and the GenBank accession number was JADDKP000000000. Analysis using the software August 3.2.1 showed that the whole genome contained 10215 encoding genes and the average length of each gene was 1513 bp. However, there was only one copy of the *FUM* gene encoding fumarase in its genome. Therefore, it was thought that there was no cytoplasmic reductive pathway for fumaric acid biosynthesis (Fig. 1). In recent years, as more and more whole-genomic DNAs in fungi have been sequenced and annotated, the whole-genomic DNA sequences have been intensively used in the fungal taxonomic classification on the basis of traditional taxonomy [25,32]. The phylogenetic tree of the whole-genomic DNA sequences from the DH177 strain, *A. pullulans* var. *aubasidani* CBS 100524 which genome has been sequenced very recently [32], *A. pullulans* P25 which could produce high level of gluconate [46] and any other *Aureobasidum* spp. and fungi showed that the DH177 strain and *A. pullulans* var. *aubasidani* CBS 100524 were localized in the same cluster and the DH177 strain was also closely related to *A. pullulans* P25 (Fig. 2). This demonstrated that the DH177 strain indeed belonged to *A. pullulans* var. *aubasidani* as described by Ref. [34].

### 3.2 Deletion of the *GOX* gene and the chemical structure identification of the product

**Fig. 3:**
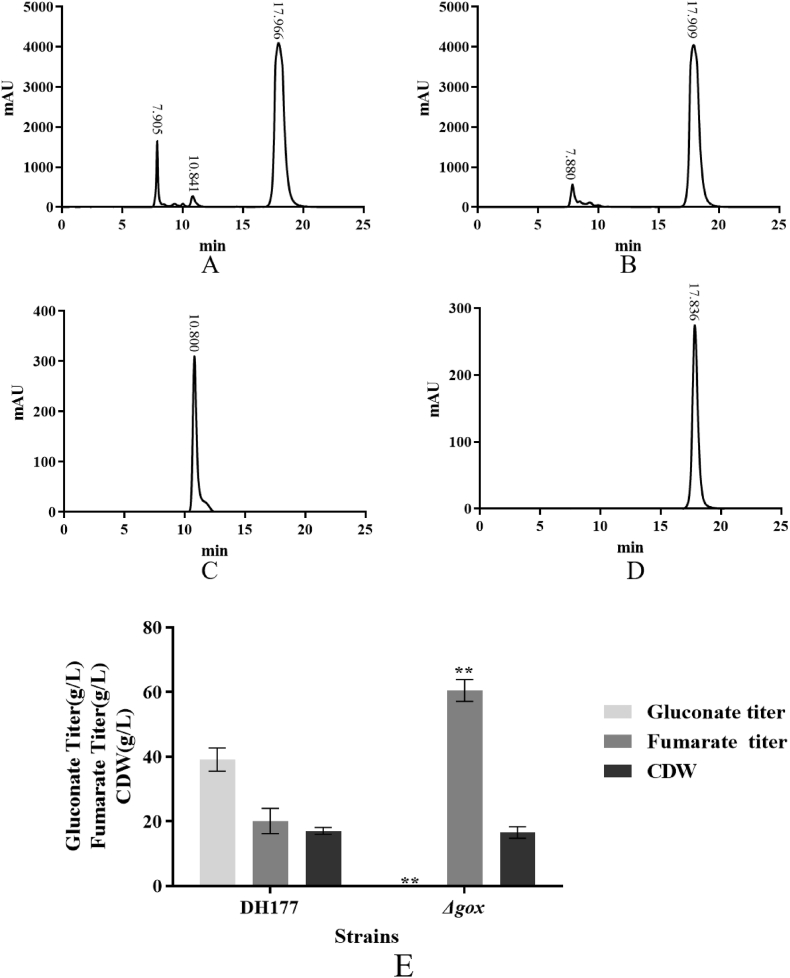
HPLC analysis of the fermentation products produced by the strain DH177 (A), the *Δgox* mutant (B), the standard gluconic acid (C) and the standard fumaric acid (D) and production of fumarate by the strain DH177 and the *Δgox* mutant (E). Data are given as mean ± SD, n = 3, *P < 0.05, **P < 0.01.

**Fig. 4:**
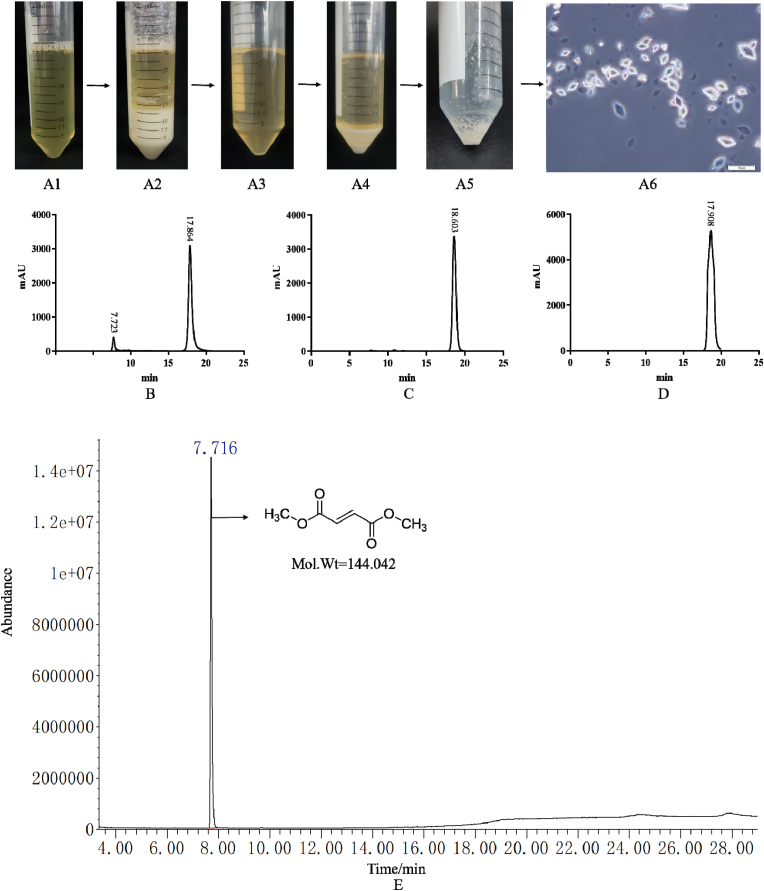
Recrystallization (A1-A6), purification and analysis of fermentation products of the mutant *Δgox*. A1-A6: the process of recrystallization (A1, the supernatant with fumarate; A2, the calcium sulfate from fumarate was precipitated by the added sulfuric acid; A3, the supernatant without calcium sulfate; A4, the recrystallized products at 4 °C; A5, the purified crystals; A6, the purified crystals observed under microscope). B: HPLC analysis of the products produced by the mutant *Δgox*; C: HPLC analysis of the recrystallized product; D: HPLC analysis of the standard fumaric acid; E: GC-MS analysis of the recrystallized product.

As shown in Table S1, the *GOX* gene encoded glucose oxidase which could transform glucose into gluconic acid. Indeed, during the cultivation of the DH177 strain, calcium gluconate (gluconate) and calcium fumarate (fumarate) were simultaneously formed in the cultures (Fig. 3). Therefore, the *GOX* gene was totally removed and the disruptant *Δgox* was obtained as described in Materials and methods (Fig. 1). The disruptant *Δgox* and its wild type strain the DH177 strain were then cultivated in the fumarate production medium and the produced gluconate, fumarate and cell mass were determined. It can be been seen from the results in Fig. 3 that gluconic acid (the peak of 10.8 min) disappeared in the products produced by the mutant *Δgox* while the DH177 strain could produce both gluconic acid (the peak of 10.8 min) and fumaric acid (the peak of 17.8 min). Then, the calcium in the products produced by the mutant *Δgox* was removed using sulfuric acid (Fig. 4A1 and 4A2). In order to further purify the products produced by the mutant *Δgox*, the products (without calcium) (Fig. 4A3) produced by the mutant *Δgox* were cooled to 4 C for crystallization and kept at this temperature for overnight (Fig. 4A4). Then, the crystals were redissolved at 80 C (Fig. 4A4). After the repeated recrystallization and resolvation, it can be clearly observed from the results in Fig. 4A5 and 4A6 that the purified crystals were formed from the products without calcium. Esterization of the crystals with methanol and analysis of the esterized products with the GC-MS showed that there was only one single peak at 7.716 min with molecular weight of 144.042 which was the molecular weight of dimethyl fumarate (Fig. 4E). This strongly demonstrated that the product (without calcium) produced by the mutant *Δgox* was fumaric acid. It also can be seen from the results in Fig. 3E that the mutant *Δgox* could produce over 60.0 g/L of fumarate.

### 3.3 The major fumaric acid biosynthesis in *A. pullulan*s var. *aubasidani* DH177

**Fig. 5:**
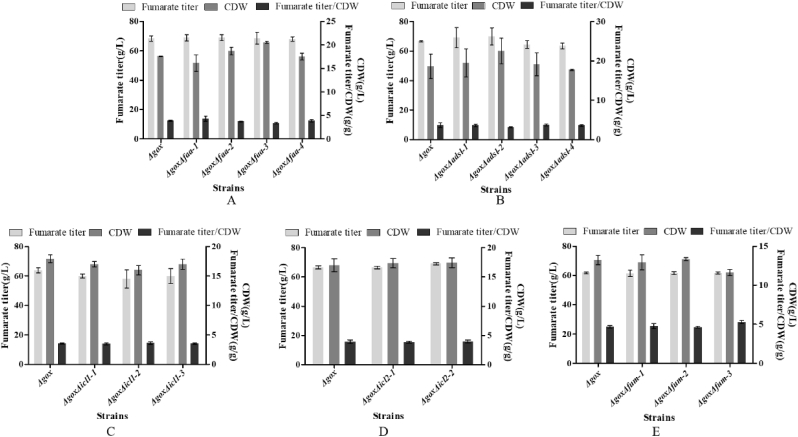
Fumarate titer and cell growth by the *Δgox* mutant and *ΔgoxΔfum* mutants (A), *ΔgoxΔadsl* mutants (B), *ΔgoxΔicl1* mutants (C), *ΔgoxΔicl2* mutants (D) and *ΔgoxΔfum* mutants (E). Data are given as mean ± SD, n = 3, *P < 0.05, **P < 0.01.

As shown in Fig. 3, the mutant *Δgox* could produce over 60.0 g/L fumarate. So, it is very important to know how it is synthesized by the mutant *Δgox.* It has been shown that in the eucaryotic cells, fumaric acid is synthesized through several metabolic pathways. The first one is the reductive branch of tricarboxylic acid (TCA) cycle and 2 mol fumaric acid/mol glucose can be obtained via 2 mol CO_2_ fixation when its cell growth is stopped. It has been reported that this pathway is considered as a major contributor for fumaric acid accumulation in *R. oryzae* [14]. The second pathway is the oxidative TCA cycle and the theoretical yield is limited to 1 mol fumaric acid/mol glucose due to the release of CO_2_. The third pathway is glyoxylate route which is suggested as a potential pathway for fumaric acid production and the theoretical yield is also limited to 1 mol/mol glucose due to the release of CO_2_, too [28,29]. In addition, the ornithine-urea cycle (OUC) (Fig. 1), purine metabolism and TTP metabolism were also involved in the synthesis of fumaric acid in fungi [14]. In order to investigate how and where the mutant *Δgox* of *A. pullulans* var. *aubasidani* synthesizes fumaric acid in its cells, the key genes *ASL*, *CPS1*, *CPS2L* and *CPS2S* in the OUC (Fig. 1), the *FAA* gene in TTP metabolism, only the *FUM* gene in the TCA cycle, the *ADSL* gene in the purine metabolism, the *SFC* gene in the mitochondrion, the two key genes *ICL1* and *ICL2* in the glyoxylate cycle were removed from the genomic DNAs of the mutant *Δgox* as described in Materials and methods. The data in Fig. 5 clearly indicated that TTP metabolism, purine metabolism, the TCA cycle and the glyoxylate cycle were not involved in the fumarate biosynthesis because the amounts of fumarate produced by all the mutants were almost the same as those produced by the mutant *Δgox*.

**Fig. 6:**
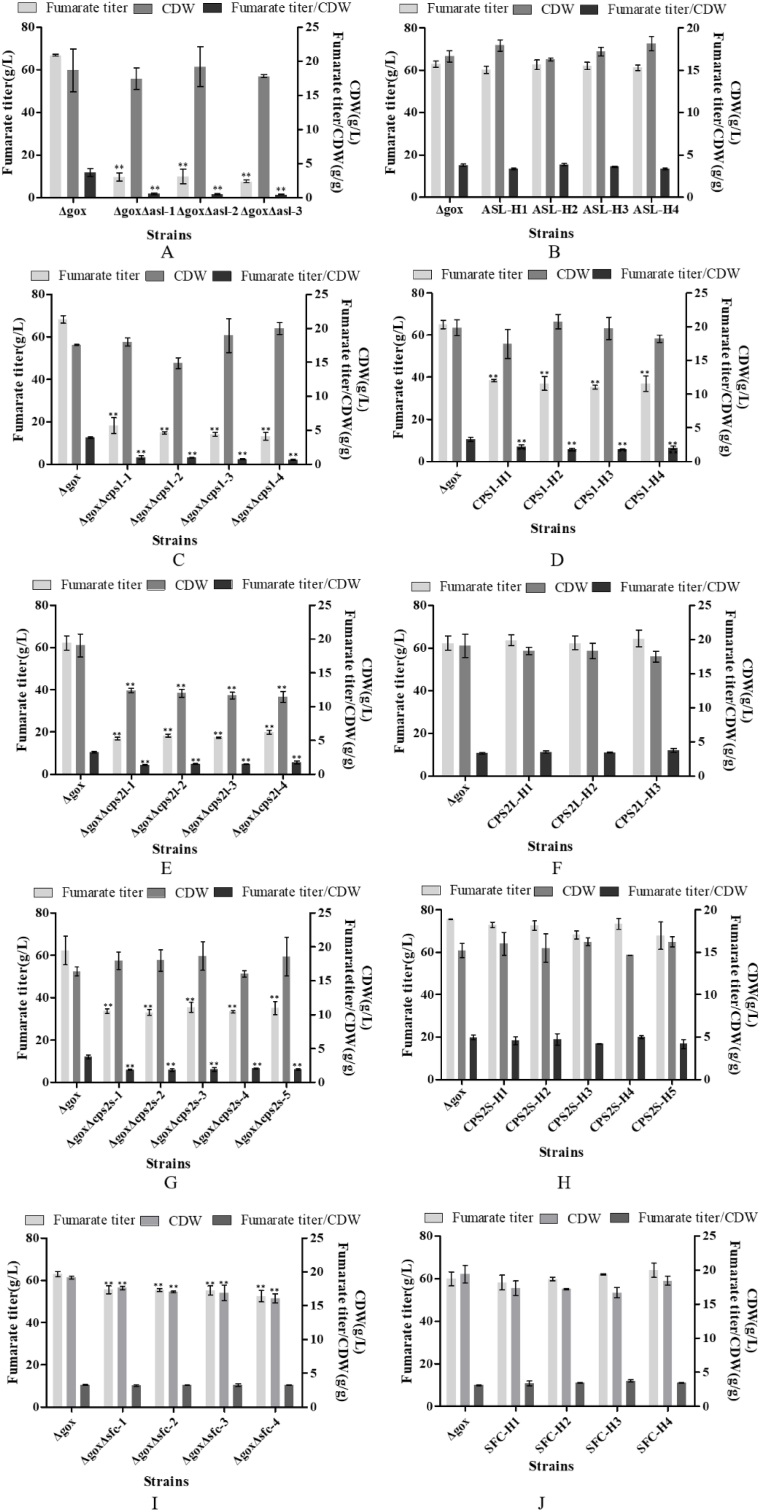
Fumarate titer and cell growth by the mutant *Δgox* and *ΔgoxΔasl* mutants (A) and their complementing strains (B); *ΔgoxΔcps1* mutants (C) and their complementing strains (D); *ΔgoxΔcps2l* mutants (E) and their complementing strains (F); *ΔgoxΔcps2s* mutants (G) and their complementing strains (H); *ΔgoxΔsfc* mutants (I) and their complementing strains (J). Data are given as mean ± SD, n = 3, *P < 0.05, **P < 0.01.

In contrast, the results in Fig. 6A, C, 6E and 6G clearly revealed that abolishment of the key *ASL* gene, *CPS1* gene, *CPS2L* gene and *CPS2S* gene in the OUC made all the mutants yield much less fumarate (about 10.0–30.0 g/L of fumarate) than that (62.0 g/L of fumarate) produced by their parent strain *Δgox*. Complementation of these genes from the OUC in the corresponding mutant restored fumarate biosynthesis (Fig. 6B, D, 6F and 6H), but could not enhance fumarate production, indicating that overexpression of these genes could not positively affect fumarate production. So, it could be concluded that the OUC controlled most of the fumarate biosynthesis in the mutant *Δgox* of *A. pullulans* var. *aubasidani.* In addition, although deletion of the *SFC* gene encoding succinate-fumarate carrier from mitochondria to cytoplasm rendered the mutants (*ΔgoxΔsfc*-1, *ΔgoxΔsfc*-2 and *ΔgoxΔsfc*-3) to produce a little less fumarate than that produced by the parent strain *Δgox* (Fig. 6I), cell growth of the mutants was also negatively affected so that all the fumarate/CDW values were almost the same (Fig. 6I). This suggested that succinate-fumarate carrier could not affect fumarate production, either. Furthermore, complementation of the *SFC* gene in the *ΔgoxΔsfc* mutants restored fumarate biosynthesis by the transformants SFC-H1, SFC-H2, SFC-H3 and SFC-H4 (Fig. 6J). Therefore, fumarate synthesized by *A. pullulans* var. *aubasidani* came mainly from OUC and the fumaric acid synthetic pathway was completely different from that in *R. oryzae* and any other fungal strains [14,28].

The data in Table S6 demonstrated that the *ASL* gene in the *ΔgoxΔasl* mutant, the *CPS1* gene in the *ΔgoxΔcps1* mutant, the *CPS2L* gene in the *ΔgoxΔcps2l* mutant and the *CPS2S* gene in the mutant *ΔgoxΔcps2s* were totally removed. Complementation of the relevant genes restored expression of some of the genes or greatly enhanced expression of some of other genes. However, complementation of the relevant genes also made some of other genes related to the OUC be downregulated. That was why the complementation could not render the ASL-H, CPS1–H, CPS2L-H and CPS2S–H strains to enhance fumarate production compared to that of the mutant *Δgox* (Table S6). This may be due to the facts that the carbon metabolism balance in the whole cells must be required. Therefore, many synthetic biology strategies must be adopted to optimize metabolism balance to further enhance fumarate biosynthesis.

### 3.4 The unique fumaric acid synthesis was controlled by the Ca^2+^ signaling pathway

**Fig. 7:**
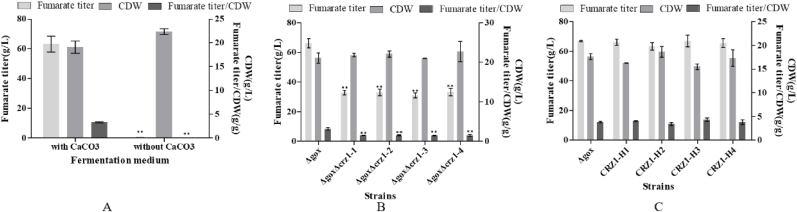
Effects of Ca^2+^ signaling pathway on fumarate biosynthesis with and without CaCO_3_ (A), effects of deletion of the *CRZ1* gene on fumarate biosynthesis (B) and effects of complementation of the *CRZ1* gene on fumarate biosynthesis (C). Data are given as mean ± SD, n = 3, *P < 0.05, **P < 0.01.

It has been confirmed that Crz1 is the key transcriptional activator in the Ca^2+^ signaling pathway [33]. Table S1 showed that the promoters of many genes related to fumaric acid biosynthesis in the OUC indeed contained the Crz1 binding site. It was found that the *Δgox* mutant could produce a large amount of fumarate only in the presence of CaCO_3_ while in the absence of CaCO_3_, fumarate production was completely stopped (Fig. 7A). Deletion of the *CRZ1* gene resulted in all the mutants (*ΔgoxΔcrz1*-1, *ΔgoxΔcrz1*-2, *ΔgoxΔcrz1*-3 and *ΔgoxΔcrz1*-4) that produced a small amount of fumarate (Fig. 7B) and complementation of the *CRZ1* gene in the disruptant *ΔgoxΔcrz1* produced the transformants (CRZ1-H1, CRZ1-H2, CRZ1-H3, and CRZ1-H4), by which fumarate production was restored compared with that in the mutant *Δgox* (Fig. 7C). So, this also could be concluded that the fumarate synthesis in the *Δgox* mutant was controlled by the Ca^2+^ signaling pathway.

**Fig. 8:**
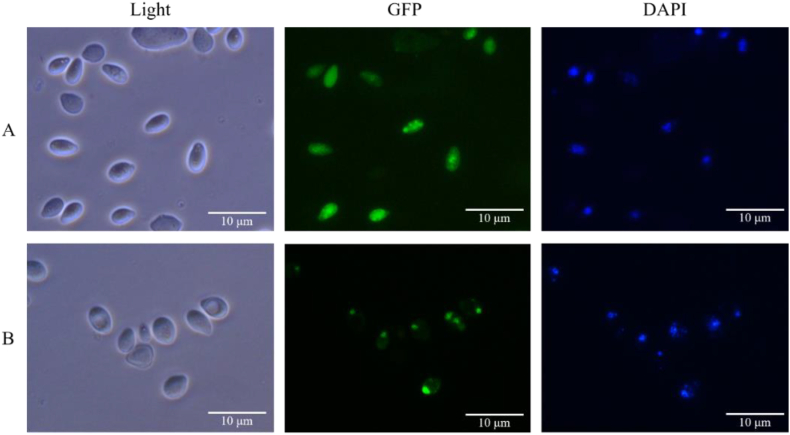
The Crz1 subcellular localization in the cells of the transformants carrying the *CRZ1*-*GFP* gene grown in the medium without CaCO_3_ (A) and with CaCO_3_ (B).

In order to further confirm this, when the transformants carrying *CRZ1*-*GFP* gene were grown in the fumarate production medium with CaCO_3_, it could be clearly observed that all the Crz1-Gfps were localized in the nuclei (Fig. 8B). In contrast, when they were grown in the fumarate production medium without CaCO_3_, all the Crz1-Gfps were distributed in the whole cells (Fig. 8A). Table S7 indeed showed that when the *Δgox* mutant was grown in the fumarate production medium with CaCO_3_, the transcriptional levels of most of the genes, such as the *ASL*, *CPS1*, *CPS2L*, *CPS2S* and *OTC* genes in the OUC were greatly enhanced compared to those of the *ASL*, *CPS1*, *CPS2L*, *CPS2S* and *OTC* genes in the *Δgox* mutant grown in the fumarate production medium without CaCO_3_. Because there were Crz1 binding sites in the promoters of the *CPS1* and *CPS2S* genes (Table S1), the transcription levels of these two genes were indeed positively regulated by Crz1. When the *CRZ1* gene was deleted, the expression of all the genes related to the OUC was down-regulated (Table S8). When the *CRZ1* gene was complemented, the expression of most of the genes related to the OUC in the transformant CRZ1-H was increased (Table S8). All the results again confirmed that the unique fumarate synthesis in the *Δgox* mutant was indeed controlled by Ca^2+^ signaling pathway via the transcriptional activator Crz1.

### 3.5 Construction of high-level fumarate producing strain by genetically engineering

**Fig. 9:**
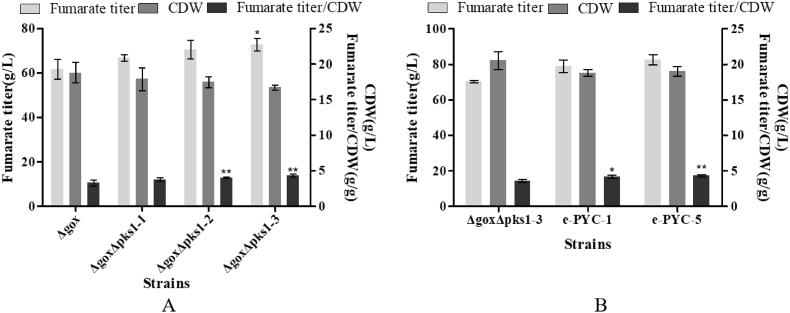
Fumarate titer and cell growth by the *Δgox* mutant, *ΔgoxΔpks1* mutants (A) and strain *ΔgoxΔpks1*, *PYC* gene over-expressed strains (B). Data are given as mean ± SD, n = 3, *P < 0.05, **P < 0.01.

Fig. 1, Fig. 6, Fig. 7, Fig. 8 had strongly confirmed that fumarate was synthesized from the OUC and its synthesis was regulated by the ca^2+^ signaling pathway. It has been well known that carbamoyl phosphate synthase (Cps) which has been confirmed to be the rate limiting enzyme in the OUC (Fig. 1), is allosterically activated by N-acetylglutamate which is synthesized from acetyl-CoA and glutamate under the action of N-acetylglutamate synthase. Therefore, we speculated that the cytoplasmic concentration of acetyl-CoA may influence the synthesis of N-acetylglutamate and in turn may influence the speed of the OUC. In our previous studies [38], it has been well documented that liamocin is a common metabolite of *Aureobasidium* spp. its biosynthesis is catalyzed by a polyketide synthase (Pks1) and acetyl-CoA is only precursor for liamocin synthesis. Therefore, blocking this pathway could increase the cytoplasmic concentration of acetyl-CoA for fumarate production. So, the *PKS1* gene was removed from the genomic DNAs in the *Δgox* mutant as described in Materials and methods. The data in Fig. 9A showed that the fumarate titer produced by the double mutant *ΔgoxΔpks1* reached 72.7 g/L while the single mutant *Δgox* only yielded 61.6 g/L of fumarate. It has been well known that the pyruvate carboxylase (Pyc) is responsible for synthesis of oxaloacetic acid by carboxylation of pyruvate in the cytoplasm [46] (Fig. 1). Then, oxaloacetic acid will be transformed into aspartic acid which is one of the substrates for argininosuccinate synthase in the OUC (Fig. 1). Many authors also stated that pyruvate carboxylase appeared to be a limiting factor, thus being a target for further metabolic engineering of fumarate producer [14,37]. Therefore, Pyc may also play an important role in fumarate biosynthesis. So, the *PYC1* gene was over-expressed in the double mutant *ΔgoxΔpks1* and the e-PYC strain (*ΔgoxΔpks1PYC1*) was obtained. It can be obviously observed from the data in Fig. 9B that the fumarate titer produced by the e-PYC strain reached to 82.5 g/L whereas the double mutant *ΔgoxΔpks1*-3 only yielded 73.6 g/L of fumarate. The genetical strategies for enhanced fumarate production are shown in Fig. S2. From the data in Table S9, it can be clearly seen that at shaking flask level, the e-PYC strains constructed in this study could produce much more fumarate than any other native strains of *R. oryzae* and the genetically engineered strains.

### 3.6 Fumarate production in the 10-l fermenter by the fed-batch fermentation

After optimization of the fumarate production medium and the inoculation size as described in Materials and methods, it was found that at flask level, the optimal compositions of the fumarate production medium were glucose 120.0 g/L, ammonium sulfate 2.0 g/L, calcium carbonate 80.0 g/L, potassium dihydrogen phosphate 0.3 g/L, magnesium sulfate heptahydrate 0.3 g/L and zinc sulfate 0.3 g/L and the inoculation size was 10% (v/v). Under these conditions, the results in Fig. S3 showed that glucose was completely consumed within 144 h and the fumarate titer reached 88.1 ± 4.3 g/L within 168 h.

**Fig. 10:**
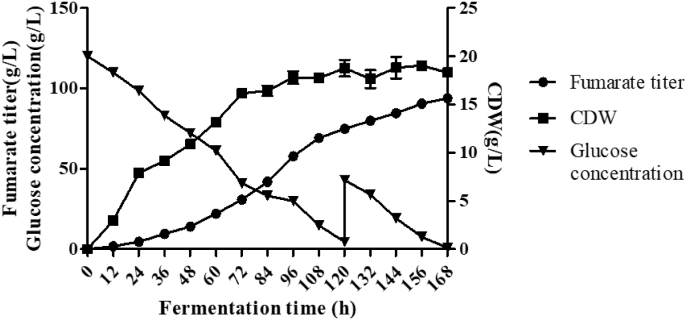
Time course of fumarate titer, cell growth of the strain e-PYC and residual glucose concentration in the 10-L fermentation. During the fed batch fermentation, additional 200 g of sterile glucose was added into the fermenter at 120 h. Data are given as mean ± SD, n = 3, *P < 0.05, **P < 0.01.

Then, the e-PYC strain was cultivated in the 10-L fermenter as described in Materials and methods and during the fed batch fermentation, additional 200 g of sterile glucose was added into the fermenter at 120 h and fumarate concentration, biomass and residual glucose concentration were measured every 12 h. It can be observed from the results in Fig. 10, the fumarate titer reached 93.9 ± 0.8 g/L within 168 h. Under these conditions, the productivity and yield reached 0.56 g/L/h and 0.63 g/g, respectively. Especially, the fumarate titer produced by the e-PYC strain was much higher than those produced by *R. oryzae* grown in the traditional stirred tank and shake flask (Table S9).

## 4 Discussion

Because there was only one copy of the *FUM* gene encoding fumarase in its genome (Fig. 1), the *FUM* gene was considered to code for only fumarase in the TCA cycle. Therefore, in this strain, there was no reductive pathway for fumaric acid biosynthesis in cytoplasm. However, fumaric acid biosynthesis in *R. oryzae* mainly comes from the reductive pathway in cytoplasm [14]. The results in Fig. 2 indicated the DH177 strain used in this study belonged to one member of *A. pullulans* var. *aubasidani* as described by Ref. [34]. It has been reported that the main exopolysaccharide produced by *A. pullulans* var. *aubasidani* strain CBS 100524 is aubasidan, rather than pullulan [32]. The aubasidans are glucans with α-1,4-D-, β-1,6-D- and β-1,3-D-glycosidic bonds and *A. pullulans* var. *aubasidani* is different from *A. pullulans* var. *pullulans* as the former had the absence of assimilation of methyl-D-glucoside and lactose [40]. However, it was interesting to note that *A. pullulans* var. *aubasidani* DH177 strain used in this study also produced fumarate in the fumarate production medium as described above. The genome of the DH177 strain contained the *GOX* gene encoding glucose oxidase (Fig. 3), Therefore, the *GOX* gene must be removed in order to make the mutant (*Δgox*) only produce fumarate (Fig. 3, Fig. 4). In our previous studies [46], *A. pullulans* P25 was also found to be able to produce high concentration of gluconate from glucose during the fermentation. The results in Fig. 3E showed that the mutant *Δgox* could produce over 60.0 g/L of fumarate while its wild type strain DH177 accumulated 32.3 g/L of fumarate in a 5-L batch fermentation [34]. This meant that the removal of the *GOX* gene could promote fumarate production. So far, the native fumarate producers have included *Zymomonas mobilis*, *Bacillus macerans*, *Thermoanaerobacter ethanolicus*, *Erwinia chrysanthemi*, S*cheffersomyces stipitis*, *Brettanomyces* or *Brett*, *Pachysolen tannophilus*, *Candida utilis*, *Rhizopus nigricans*, *R. arrhizus*, *R. oryzae*, *R. formosa*, *Cunninghamella*, *Cirnella* spp., *Penicillum griseofulvum*, *Aspergillus glaucus*, and *Caldariomycels fumago* [3, 5]. Recently, the genetically engineered strains of *E. coli*, *S. cerevisiae*, *T. glabrata*, *S. stipites* and *R. oryzae* also have been used for fumarate production. Among them, *R. oryzae* is the best and major fumarate producer. However, all the engineered strains had lower ability to produce fumarate than the wild type strain *R. oryzae* [14,28]. Furthermore, there are still many major obstacles for the commercialization of fumaric acid bio-production by *R. oryzae* [21] as stated in the Introduction section. So, this is the first time to get enough evidence to confirm that the *Δgox* mutant of *A. pullulans* var. *aubasidani* could synthesize high level of fumarate (Fig. 3E). It has been reported that *A. pullulans* var. *aubasidani* strain produced only aubasidan, rather than pullulan [40]. However, it was found that when the wild type strain DH177 was cultivated in the fumarate production medium, no exopolysaccharides were formed (data not shown). As shown above, as a yeast-like fungus and fumarate producer, *A. pullulans* var. *aubasidani* has many unique merits over any other yeasts and fungal strains, especially *R. oryzae* [14,28]. For example, the culture of *A. pullulans* var. *aubasidani* had major yeast cells*,* could not form clumps and pellets during cultivation (data not shown), its genome could be easily edited (Fig. 5, Fig. 6) by continuous deletion and expression of the target genes.

Fig. 5, Fig. 6 showed that only OUC was involved in fumarate biosynthesis in the *Δgox* mutant. It is true that the glyoxylate cycle cannot be involved in fumaric acid production because its key enzymes Icl1 and Icl2 (Table S1) are easily repressed in the presence of high glucose concentrations in the fumarate production medium [31]. It has well known that fumaric acid is an important intermediate of the TCA cycle, but the fumaric acid generated during the oxidative pathway is not accumulated as it is consumed for ATP production, cell growth and maintenance [14]. Although expression of the cytoplasmic Fum from *R. oryzae* could enhance fumarate generation [14], the *FUM* gene was considered not to exist in the genome of *A. pullulans* var. *aubasidani* DH177 as mentioned above. Therefore, fumarate synthesized by *A. pullulans* var. *aubasidani* came mainly from OUC and the fumaric acid synthetic pathway was completely different from that in *R. oryzae* and any other fungal strains [14,28]. It has been reported that the enhanced expression of argininosuccinate lyase (Asl) from the OUC and decreased expression of adenylosuccinate lyase (Adsl) from the purine nucleotide cycle could enable the engineered strain of *T. glabrata* to produce very low titer (5.6 g/L) of fumarate [7]. In addition, the C4-dicarboxylic acids transporter in *Schizosaccharomyces pombe* is involved in effective export of l-malic acid, fumaric acid, and succinic acid from cytoplasm to medium [41] and the succinate-fumarate transporter (*SFC*) encoded by *ACR1* gene in *S. cerevisiae* is responsible for transport of fumaric acid from mitochondria into cytoplasm [26]. In contrast, the accumulation of main fumaric acid occurs via the reductive TCA pathway, as observed in the case of *R. oryzae*. However, the cytosolic fumarase mainly catalyzes the conversion of fumaric acid to l-malic acid in any other fungi. Therefore, this was the first time to get the evidence to show that the Asl activity which catalyzed release of fumaric acid from argininosuccinate in the OUC (Fig. 1) was responsible for only fumarate production by *A. pullulans* var. *aubasidani.* It has been regarded that Asl in the OUC serve as a bridge between carbon metabolism and nitrogen metabolism in fungal cells [7]. As shown in Fig. 1, in the presence of CaCO_3_, the synthesized fumaric acid could be efficiently transformed into fumarate so that the bridge between carbon metabolism and nitrogen metabolism in the fungal cells was disconnected. That was why accumulation of high level of fumarate could occur by *A. pullulans* var. *aubasidani* used in this study. Meanwhile, HCO_3_^2−^ released from CaCO_3_ in the medium used in this study might also take part in the first reaction by the Cps which was a rate limiting reaction in the OUC (Fig. 1). Therefore, according to Fig. 1, mol of glucose *theoretically* can produce 2 mols of oxaloacetate by fixation of 2 mols of CO_2_ through the glycolysis pathway under catalysis of pyruvate carboxylase (Pyc). Although OUC fixes 1 mol of CO_2_ from CO_3_^2−^ released from the added CaCO_3_, 1 mol of urea containing one carbon is lost during the ornithine-urea cycle, leading to no net carbon fixation in the cycle. In fact, the maximal theoretical yield will never be obtained because some of the added glucose is needed to be metabolized to produce ATP, cell components and cell growth so that during the fed-batch fermentation, the productivity and yield only reached 0.56 g/L/h and 0.63 g/g of glucose (Fig. 10). However, as mentioned above, the reductive pyruvate carboxylation also has a maximal theoretical yield of 2 mol of fumaric acid by fixation of 2 mol CO_2_ only when nitrogen becomes limiting and the growth phase stops while TCA cycle and glyoxylate cycle have a maximal theoretical yield of only 1 mol of fumaric acid per mole of glucose consumed with release of 2 mol CO_2_ and without fixation of any CO_2_, respectively [29]. So, here we strongly confirmed the OUC-mediated fumaric acid biosynthesis pathway in *A. pullulans* var. *aubasidani* was a green and eco-friendly process for the global sustainable development and carbon neutrality. In order to make *E. coli* produce fumarate, the mammalian OUC was introduced into *E. coli*. However, the final strain only yielded a fumarate titer of 1.3 g/L [44]. Overexpression levels of the enzymes Asl from the OUC and Adsl from the purine nucleotide cycle in *T. glabrata* gave the engineered strain to produce a fumarate titer of only 5.6 g/L [7]. This meant that the OUC in *E. coli* and *T. glabrata* failed to make contribution to high fumarate production. However, the mutant *Δgox* obtained in this study could produce over 60.0 g/L of fumarate (Fig. 3E). All the results in Fig. 7, Fig. 8 and in Tables S1, S7 and S8 confirmed that the unique fumarate synthesis was indeed controlled by Ca^2+^ signaling pathway via the transcriptional activator Crz1. In our previous study, it was also confirmed that the biosynthesis of polymalate (PMA) in *A. melanogenum* is also regulated by the Ca^2+^ signaling pathway via the transcriptional activator Crz1 [33]. From Fig. 1, it also can be seen that HCO_3_^−1^ released from CaCO_3_ is required for the reaction under catalysis of CpsI and CpsII. That was why the added CaCO_3_ was required for PMA and fumarate production (Fig. 7A).

From the data in Table S9 and Fig. S3, it can be clearly observed that at shaking flask level, the e-PYC strain constructed in this study could produce much more fumarate than any other native strains of *R. oryzae* and the genetically engineered strains. For example, 56.5 g/L of fumarate was produced by the wild type strain *R. oryzae* while only 41.5 g/L, 25.0 g/L, 5.64 g/L, 9.2 g/L and 4.7 g/L of fumarate were produced by engineered *E. coli* EF02(pSCppc) [19], *R. oryzae* ppc [12], *S. cerevisiae* FMME 006 *FUM1* + *RoPYC* + *RoMDH* + *RoFUM1* [37], *T. glabrata* (*Δade12* for denylosuccinate synthetase)–PMS–P160A [5] and *S. stipites* [36], respectively. Furthermore, all the results mentioned above demonstrated that it was very easy to edit the genomic DNAs of *A. pullulans* var. *aubasidani* DH177 and operate cell growth of it and its mutants in the bioreactor. In contrast, it is very difficult to operate the growth of the filamentous *Rhizopus* spp. in the bioreactor and to edit genomic DNAs of them [14]. This meant that the yeast-like fungus used in this study had high potential in bioproduction of fumarate. Finally, the strain e-PYC constructed in this study could produce 88.1 ± 4.3 g/L of fumarate at a flask level and 93.9 ± 0.8 g/L of fumarate during the fed-batch fermentation (Figs. S3 and 10). According to the data in Table S9, this fumarate titer was the highest level compared to that produced by most of any other strains, especially *R. oryzae* and its genetically engineered strains grown at the flask level and the 10-liter fermenter. This again demonstrated that the yeast-like fungus used in this study indeed had very high potential in bioproduction of fumarate although the bioprocess still remained less economically competitive compared with the traditional petrochemical method.

## 5 Conclusions

In this study, it was found that the mutants of *A. pullulans* var. *aubasidani* DH177 was better than *R. oryzae* and any other fungi for bioproduction of fumarate because it was easy to genetically edit genomes of them, operate their cell growth in the fermenter and their fumarate titer was much higher than that produced by any other fungi. The whole genome mutation showed only the OUC was involved in fumarate biosynthesis which was controlled by Ca^2+^-signaling pathway. Removal of both the *GOX* gene and the *PKS1* gene and overexpression of the *PYC1* gene made the e-PYC strain yield 93.9 ± 0.8 g/L of fumarate and have the productivity and the yield of 0.56 g/L/h and 0.63 g/g during the fed batch fermentation. These showed that the yeast-like fungus used in this study indeed had very high potential in green and eco-friendly bioproduction of fumarate for the global sustainable development and carbon neutrality.

## CRediT authorship contribution statement

**Xin Wei:** Methodology, Data curation, Formal analysis, Investigation, and. **Miao Zhang:** Methodology, Data curation, Formal analysis, Investigation. **Guang-Yuan Wang:** Writing – original draft, Investigation, and. **Guang-Lei Liu:** Writing – original draft, and, Investigation. **Zhen-Ming Chi:** Writing – review & editing, Project administration, Resources, Funding acquisition, and. **Zhe Chi:** Writing – review & editing, Project administration, Resources, Funding acquisition.

## Declaration of competing interest

The authors declare that there is no conflict of interest.
