# Supplementary material for: The ornithine-urea cycle involves fumaric acid biosynthesis in Aureobasidium pullulans var. aubasidani, a green and eco-friendly process for fumaric acid production
Source: Synth Syst Biotechnol. 2022 Oct 19;8(1):33–45. doi: 10.1016/j.synbio.2022.10.004 (PMC9647333; doi:10.1016/j.synbio.2022.10.004)
Supplement: Multimedia component 1 [file mmc1.doc]

**Table S1 The genes cloned, characterized and used in this study**

| Gene | Function | Pathways controlled by the genes | GenBank Accession No. | Crz1 binding site |
| --- | --- | --- | --- | --- |
| *GOX* | Glucose oxidase | Gluconate synthesis | MZ734615 | / |
| *PKS1* | Highly reducing PKS | 3,5 -dihydroxydecanoic acid | MZ734618 | GAGGCGCA (-857 bp)  GAGCCC (-99 bp) |
| *CRZ1* | Transcriptional activator | Ca^2+^ signaling pathway | MZ734617 | GGGGCGCA (-793 bp) |
| *PYC* | Pyruvate carboxylase | Cytoplasmic reduction pathway | MZ734619 | / |
| *FAA* | Fumarylacetoacetase | TTP metabolism | OK052605 | / |
| *ADSL* | Adenylosuccinate lyase | Purine metabolism | OK052608 | / |
| *ASL* | Argininosuccinate lyase | OUC | MZ734616 | / |
| *CPS1* | Carbamoyl phosphate synthase 1 | OUC | OK012323 | GGGGCTG (-515 bp) |
| *CPS2L* | Carbamoyl phosphate synthase Ⅱ large subunit | OUC and pyrimidine metabolism | OK012324 | / |
| *CPS2S* | Carbamoyl phosphate synthase Ⅱ small subunit | OUC and pyrimidine metabolism | OK012325 | GAGCCC (-733 bp) |
| *OTC* | Ornithine transcarbamoylase | OUC | OM047205 | / |
| *ASS* | Argininosuccinate synthase | OUC | OM047206 | / |
| *ARG* | Arginase | OUC | OM047207 | / |
| *FUM* | Fumarase | TCA cycle | OK052609 | / |
| *SFC* | Succinate-fumarate carrier | TCA cycle | OK012322 | / |
| *ICL1* | Isocitrate lyase1 | Glyoxylate cycle | OK052606 | / |
| *ICL2* | Isocitrate lyase2 | Glyoxylate cycle | OK052607 | / |
