# Supplementary material for: The ornithine-urea cycle involves fumaric acid biosynthesis in Aureobasidium pullulans var. aubasidani, a green and eco-friendly process for fumaric acid production
Source: Synth Syst Biotechnol. 2022 Oct 19;8(1):33–45. doi: 10.1016/j.synbio.2022.10.004 (PMC9647333; doi:10.1016/j.synbio.2022.10.004)
Supplement: Multimedia component 2 [file mmc2.doc]

**Table S2 The PCR primers for the construction of the knock-out vectors and the knock-in vectors**

| Primers | Sequences (5’-3’) |
| --- | --- |
| ASL-F | ACGCGTATGGCAGGTGCACCTAGCA (*Mlu*Ⅰ) |
| ASL-R | CTGCAGTTACTTCTTCTCGATGGTCTGCTT (*Pst*Ⅰ) |
| CPS1-F | ACTAGTATGGCTGGCCACACTGGA (*Spe*Ⅰ) |
| CPS1-R | ACGCGTTTATGATGCCATGACCAA (*Mlu*Ⅰ) |
| CPS2L-F | ACGCGTATGGCTTTCTCCAGGCGT (*Mlu*Ⅰ) |
| CPS2L-R | CTGCAGTTACAGAGCGCGGCCGC (*Pst*Ⅰ) |
| CPS2S-F | ACGCGTATGATCCCCACCATCTTC (*Mlu*Ⅰ) |
| CPS2S-R | CTGCAGTCAAGCAACGACGGGCTC (*Pst*Ⅰ) |
| SFC-F | GAGCTCATGTCGGCAAAATCAAC (*Sac*Ⅰ) |
| SFC-R | GTCGACTCACTCTTCGTAACTTCC (*Sal*Ⅰ) |
| CRZ1-F | CTTAAGATGAGCAACCAAGGCCAG(*Afl*Ⅱ) |
| CRZ1-R | GAGCTCTCGCTCCATGTCACTCAT(*Sac*I) |
| GFP-F | GAGCTCGGTGGCTCTGGCGGCGGC(*Sac*I) |
| GFP-R | GTCGACTTACTTGTACAGCTCGTC(*Sal*I) |
| PYC-F | ACTAGTATGTCGGACTTTGCAGCT(*Spe*I) |
| PYC-R | CTTAAGTTATTTGACGATCTTGCA(*Afl*Ⅱ) |
| GOX-5F | CTGCAGTTCTCTCTTCTGTTGGCT (*Pst*I) |
| GOX-5R | GTCGACGAGGATGTATGCAGTGTA (*Sal*I) |
| GOX-3F | GGATCCTCCTTGGCAATGCATCCT (*Bam*HI) |
| GOX-3R | GAATTCCCTCTCTAATCATAACGG (*Eco*RI) |
| CRZ1-5F | CTGCAGCTCCTGGCTATCATGGTA (*Pst*I) |
| CRZ1-5R | GTCGACTCGAAGGAGGAGCAAAAT (*Sal*I) |
| CRZ1-3F | GGATCCGCATACAATCTACGCTCC (*Bam*HI) |
| CRZ1-3R | GAATTCCTCGCTAAGATCTCCCCA (*Eco*RI) |
| PKS1-5F | CTGCAGATCAGCTACGCTCGAACC (*Pst*I) |
| PKS1-5R | GTCGACCTGTGAAGTGCCACCAAT (*Sal*I) |
| PKS1-3F | GGATCCGTAACGTCGGTCATCTCG (*Bam*HI) |
| PKS1-3R | GAATTCCGACAAGGTAGATGGGAG (*Eco*RI) |
| ASL-5F | CTGCAGCTGAAAGACACG (*Pst*I) |
| ASL-5R | GTCGACAGTCGCACGAAT (*Sal*I) |
| ASL-3F | GGATCCAACGACCTTGAGAGAC (*Bam*HI) |
| ASL-3R | GAATTCGCTTGTCCATAGGAGTGC (*Eco*RI) |
| CPS1-5F | CTGCAGACTGTCATCACCACCGAG (*Pst*I) |
| CPS1-5R | GTCGACTCTTCATAGCAGAGCCGA (*Sal*I) |
| CPS1-3F | GGATCCGTGTGGTTGTCCAGTGAG (*Bam*HI) |
| CPS1-3R | GAATTCTTGATGTCGTTGTCCT (*Eco*RI) |
| CPS2L-5F | CTGCAGTTCGTCAGAGCCAGCAGC (*Pst*I) |
| CPS2L-5R | GTCGACTGTCCTCACTGGTCTTC (*Sal*I) |
| CPS2L-3F | GGATCCACCGTAGATGGTTGGCTG (*Bam*HI) |
| CPS2L-3R | GAATTCGAAGTAGAGCTTGTCGGC (*Eco*RI) |
| CPS2S-5F | CTGCAGACCAAGGCTTTGATGACC (*Pst*I) |
| CPS2S-5R | GTCGACGCTAATGTAGTCTGGCA (*Sal*I) |
| CPS2S-3F | GGATCCGCTCTGATTGACTGCGGT (*Bam*HI) |
| CPS2S-3R | GAATTCGAGCAAGTCAACGAGCA (*Eco*RI) |
| SFC-5F | CTGCAGTGCTTGAGCCAGAAGAG (*Pst*I) |
| SFC-5R | GTCGACAAATGGGGTAGCTGCGA (*Sal*I) |
| SFC-3F | GGATCCTAGCGGCAGGAGTTACAG (*Bam*HI) |
| SFC-3R | GAATTCTCCTGGAGCCATTTCT (*Eco*RI) |
| ADSL-5F | CTGCAGATCATGCCTCACCCTAC (*Pst*I) |
| ADSL-5R | GTCGACTGGACCCAAGCCATAACA (*Sal*I) |
| ADSL-3F | GGATCCGCTATCCGCCGTATTGAC (*Bam*HI) |
| ADSL-3R | GAATTCTAAGTGACAAACGCCAGC (*Eco*RI) |
| FAA-5F | CTGCAGCTGGTTAGGAATCTGGT (*Pst*I) |
| FAA-5R | GTCGACGGTCGTTGACTATTGTG (*Sal*I) |
| FAA-3F | GGATCCAGCTCGACATCGAAATCG (*Bam*HI) |
| FAA-3R | GAATTCATTCTCCAAAGCCCACT (*Eco*RI) |
| ICL1-5F | CTGCAGACGCCGAAGAGCAACAGT (*Pst*I) |
| ICL1-5R | GTCGACGGAGCTTGGTCCTCGATA (*Sal*I) |
| ICL1-3F | GGATCCTCGTTCAACTGGAAGACC (*Bam*HI) |
| ICL1-3R | GAATTCCTTGTATCCACTCTCTC (*Eco*RI) |
| ICL2-5F | CTGCAGAGGCAAGGTTCTGGTTCC (*Pst*I) |
| ICL2-5R | GTCGACTCGAGCCATAGCAGATC (*Sal*I) |
| ICL2-3F | GGATCCGCTATCTGGACGGCATTC (*Bam*HI) |
| ICL2-3R | GAATTCTCAGAATGGGTAGTGCT (*Eco*RI) |

The underlined bases are the target sites of the DNA restriction enzymes in ( ).
