# Supplementary material for: The ornithine-urea cycle involves fumaric acid biosynthesis in Aureobasidium pullulans var. aubasidani, a green and eco-friendly process for fumaric acid production
Source: Synth Syst Biotechnol. 2022 Oct 19;8(1):33–45. doi: 10.1016/j.synbio.2022.10.004 (PMC9647333; doi:10.1016/j.synbio.2022.10.004)
Supplement: Multimedia component 3 [file mmc3.doc]

**Table S3** The software used in this study

| Applications | Website |
| --- | --- |
| Analysis of the conserved domains of the proteins | https://www.ncbi.nlm.nih.gov/ |
| Analysis of signal peptide | http://cbs.dtu.dk/services/SignalP/ |
| Analysis of identification of proteins | http://www.ebi.ac.uk/interpro/ |
| Analysis of transmembrane domains of protein | http://www.cbs.dtu.dk/services/TMHMM/ |
| Analysis of subcellular localization of proteins | https://psort.hgc.jp/form2.html |
