# Supplementary material for: The ornithine-urea cycle involves fumaric acid biosynthesis in Aureobasidium pullulans var. aubasidani, a green and eco-friendly process for fumaric acid production
Source: Synth Syst Biotechnol. 2022 Oct 19;8(1):33–45. doi: 10.1016/j.synbio.2022.10.004 (PMC9647333; doi:10.1016/j.synbio.2022.10.004)
Supplement: Multimedia component 4 [file mmc4.doc]

**Table S5** The primers used in the fluorescent real-time PCR assay

| Primers | Sequences (5’-3’) |
| --- | --- |
| Actin-se | CATCAACCCCAAGTCCAACC |
| Actin-an | CCTTCGTAGATGGGGACA |
| CPS1-se | TTGCCTCGTCGCTCTTCA |
| CPS1-an | CAGTCTGCTCGTTGGTGGA |
| CPS2L-se | CCCGATGGTATCTTCCTTTC |
| CPS2L-an | GGGTAGCCGACCTTCTCA |
| CPS2S-se | AGTCGCCCAACATCCAGG |
| CPS2S-an | CGCACCACTCGCTCAAAC |
| OTC-se | TGTTTGACTTGGCTATTGGTG |
| OTC-an | CAGCGTCTTGGATGATTTGA |
| ASS-se | AAAAGGTCTGTCTCGCCTACTCTG |
| ASS-an | TCGGCACCAATCTTGAGGG |
| ASL-se | ATCCCTTGATGACTGCCTACA |
| ASL-an | CCTCCACTCCTCTTCCACCT |
| ARG-se | AGCCCAAGTTCCTTCCCTC |
| ARG-an | ACCTCGCCGTCGTAGTGAA |
