# Supplementary material for: The ornithine-urea cycle involves fumaric acid biosynthesis in Aureobasidium pullulans var. aubasidani, a green and eco-friendly process for fumaric acid production
Source: Synth Syst Biotechnol. 2022 Oct 19;8(1):33–45. doi: 10.1016/j.synbio.2022.10.004 (PMC9647333; doi:10.1016/j.synbio.2022.10.004)
Supplement: Multimedia component 5 [file mmc5.doc]

**Table S6** Transcriptional levels of the genes of the OUC in different disruptants and transformants

Data are given as mean ± SD, n=3, ^*^ *P* < 0.05, ^**^ *P* < 0.01. * means difference; ** means significant difference

| Gene | *CPS1* | *CPS2L* | *CPS2S* | *OTC* | *ASS* | *ASL* | *ARG* |
| --- | --- | --- | --- | --- | --- | --- | --- |
| *Δgox* (%) | 100.0 | 100.0 | 100.0 | 100.0 | 100.0 | 100.0 | 100.0 |
| *ΔgoxΔasl* (%) | 44.7 ± 5.5^**^ | 153.1 ± 10.0^**^ | 248.9 ± 9.5^**^ | 54.5 ± 2.7^**^ | 103.4 ± 5.1 | 0.0 ± 0.0 | 31.9 ± 2.6^**^ |
| *ΔgoxΔcps1* (%) | 0.9 ± 0.9^**^ | 293.5 ± 11.1^**^ | 261.7 ± 6.4^**^ | 84.7 ± 9.2^*^ | 154.8 ± 12.0^**^ | 32.8 ± 2.6^**^ | 21.0 ± 0.5^**^ |
| *ΔgoxΔcps2l* (%) | 199.6 ± 24.9^**^ | 0.0 ± 0.0^**^ | 2448.3 ± 199.7^**^ | 120.1 ± 6.2^**^ | 1175.1 ± 108.8^**^ | 115.6 ± 9.9^*^ | 60.4 ± 5.8^**^ |
| *ΔgoxΔcps2s* (%) | 101.5 ± 12.6 | 839.3 ± 73.6^**^ | 0.0 ± 0.0^**^ | 210.6 ± 31.2^**^ | 559.2 ± 22.7^**^ | 193.5 ± 17.1^**^ | 80.4 ± 7.4^**^ |
| ASL-H (%) | 56.5 ± 3.9^**^ | 85.6 ± 4.6^**^ | 110.0 ± 7.4 | 81.1 ± 7.2^**^ | 129.1 ± 9.9^**^ | 5010.1 ± 450.6^**^ | 87.0±7.4^**^ |
| CPS1-H (%) | 106.5 ± 4.1 | 1198.1 ± 20.4^**^ | 0.1 ± 0.0^**^ | 138.3 ± 9.8^**^ | 780.3 ± 41.9^**^ | 171.9 ± 14.2^**^ | 56.5 ± 3.6^**^ |
| CPS2L-H (%) | 86.1 ± 3.6^**^ | 543.9 ± 43.9^**^ | 117.7 ± 8.9^**^ | 61.1 ± 1.9^**^ | 107.1 ± 8.6 | 78.9 ± 6.0^**^ | 62.7 ± 7. 5^**^ |
| CPS2S-H (%) | 86.6 ± 3.0^**^ | 108.2 ± 18.8 | 278.3 ± 31.1^**^ | 97.9 ± 11.3 | 89.0 ± 3.9^**^ | 93.0 ± 9.3 | 105.0 ± 12.3 |
