# Supplementary material for: The ornithine-urea cycle involves fumaric acid biosynthesis in Aureobasidium pullulans var. aubasidani, a green and eco-friendly process for fumaric acid production
Source: Synth Syst Biotechnol. 2022 Oct 19;8(1):33–45. doi: 10.1016/j.synbio.2022.10.004 (PMC9647333; doi:10.1016/j.synbio.2022.10.004)
Supplement: Multimedia component 6 [file mmc6.doc]

**Table S7** Transcriptional levels of the genes of the OUC in the mutant *Δgox* grown in the presence or absence of CaCO_3_

Data are given as mean ± SD, n=3, ^*^ *P* < 0.05, ^**^ *P* < 0.01. * means difference; ** means significant difference

| Genes | without CaCO_3_ (%) | with CaCO_3_ (%) |
| --- | --- | --- |
| *CPS1* | 100.0 | 226.7 ± 9.1^**^ |
| *CPS2L* | 100.0 | 286.6 ± 31.0^**^ |
| *CPS2S* | 100.0 | 345.9 ± 9.5^**^ |
| *OTC* | 100.0 | 154.0 ± 1.6^**^ |
| *ASS* | 100.0 | 91.3 ± 2.7^**^ |
| *ASL* | 100.0 | 322.0 ± 29.7^**^ |
| *ARG* | 100.0 | 24.2 ± 1.6^**^ |
