# Supplementary material for: The ornithine-urea cycle involves fumaric acid biosynthesis in Aureobasidium pullulans var. aubasidani, a green and eco-friendly process for fumaric acid production
Source: Synth Syst Biotechnol. 2022 Oct 19;8(1):33–45. doi: 10.1016/j.synbio.2022.10.004 (PMC9647333; doi:10.1016/j.synbio.2022.10.004)
Supplement: Multimedia component 7 [file mmc7.doc]

**Table S8** Transcriptional levels of the genes of the OUC in the single mutant *Δgox,* the double mutant *ΔgoxΔcrz1* and the complementing strain CRZ1-H

Data are given as mean ± SD, n=3, ^*^ *P* < 0.05, ^**^ *P* < 0.01. * means difference; ** means significant difference

| Genes | *Δgox* (%) | *ΔgoxΔcrz1* (%) | CRZ1-H (%) |
| --- | --- | --- | --- |
| *CPS1* | 100.0 | 43.3 ± 2.6^**^ | 136.4 ± 4.5^**^ |
| *CPS2L* | 100.0 | 67.5 ± 5.4^**^ | 116.2 ± 5.4^**^ |
| *CPS2S* | 100.0 | 54.7 ± 2.8^**^ | 132.9 ± 9.3^**^ |
| *OTC* | 100.0 | 35.8 ± 4.7^**^ | 44.5 ± 6.7^**^ |
| *ASS* | 100.0 | 96.2 ± 8.0 | 144.0 ± 4.6^**^ |
| *ASL* | 100.0 | 44.3 ± 2.3^**^ | 77.6 ± 4.0^**^ |
| *ARG* | 100.0 | 73.8 ± 2.7^**^ | 76.0 ± 3.2^**^ |
