# Supplementary material for: The ornithine-urea cycle involves fumaric acid biosynthesis in Aureobasidium pullulans var. aubasidani, a green and eco-friendly process for fumaric acid production
Source: Synth Syst Biotechnol. 2022 Oct 19;8(1):33–45. doi: 10.1016/j.synbio.2022.10.004 (PMC9647333; doi:10.1016/j.synbio.2022.10.004)
Supplement: Multimedia component 8 [file mmc8.doc]

**Table S9** Production of fumarate from glucose by different strains of *R. oryzae* grown in the traditional bioreactor

| Strains | Bioreactor | Mutation strategy | Substrate | Titer  (g/L) | | Productivity  (g/L/h) | Yield  (g/g) | References | |
| --- | --- | --- | --- | --- | --- | --- | --- | --- | --- |
| *R. oryzae* ATCC 20344 | Shake flask | Native strain | Glucose | 30.2 | | 0.19 | 0.28 | [27] | |
| *R. oryzae* ATCC 20344 | Stirred tank | Native strain | Glucose | 58.7 | | 0.66 | 0.60 | [11] | |
| *R. oryzae* ATCC 20344 | Stirred tank | Native strain | Glucose | 65.0 | | 0.9 | 0.65 | [14] | |
| *R. oryzae* ATCC 20344 | Stirred tank | Native strain | Glucose | 36.0 | | 0.9 | 0.60 | [47] | |
| *R. oryzae* ME-F12 | Stirred tank | Native strain | Glucose | 56.2 | | 1.3 | 0.54 | [13] | |
| *R. oryzae* ATCC  20344 | Immobilized fungi | Native strain | Glucose | 40.1 | | 0.42 | 0.75 | [23] | |
| *R. oryzae* ZJU11 | Shake flask | UV | Glucose | | 57.4 | / | / | | [18] |
| *R. oryzae* RUR709 | Shake flask | UV and γ-rays | Glucose | | 26.2 | 0.22 | 0.32 | | [17] |
| *R. oryzae* ME-UN-8 | Stirred tank | UV | Glucose | | 52.70 | / | / | | [13] |
| *R. oryzae* ZJU11 | Stirred tank | UV | Glucose | | 41.10 | 0.37 | 0.48 | | [18] |
| *R. oryzae* NRRL 1526 | The immobilized *R. oryzae* | Native strain | Glucose | | 27.0 | 0.19 | 0.27 | | [29] |
| *R. oryzae* RUR709 | Stirred tank | UV and γ-rays | Glucose | | 32.10 | 0.32 | 0.45 | | [17] |
| *R. oryzae* PEPC | Shake flask | Genetically engineered | Glucose | | 25. 0 | 0.26 | 0.78 | | [42] |
| e-PYC of *A. pullulans* var. *aubasidani* DH177 | Stirred tank | Genetically engineered | Glucose | | 93.9 | 0.56 | 0.63 | | This study |
