# Supplementary material for: The ornithine-urea cycle involves fumaric acid biosynthesis in Aureobasidium pullulans var. aubasidani, a green and eco-friendly process for fumaric acid production
Source: Synth Syst Biotechnol. 2022 Oct 19;8(1):33–45. doi: 10.1016/j.synbio.2022.10.004 (PMC9647333; doi:10.1016/j.synbio.2022.10.004)
Supplement: Multimedia component 9 [file mmc9.doc]

**Table S4 Different disruptants and transformants of *A. pullulans* var. *aubasidani* DH177 (wild type strain) used in this study**

| Strain | Genotype | Reference |
| --- | --- | --- |
| DH177 | Wild type | Wang et al., 2018 |
| *Δgox* | *Δgox* | This study |
| *ΔgoxΔpks1* | *ΔgoxΔpks1* | This study |
| *ΔgoxΔfaa* | *ΔgoxΔfaa* | This study |
| *ΔgoxΔadsl* | *ΔgoxΔadsl* | This study |
| *ΔgoxΔfum* | *ΔgoxΔfum* | This study |
| *ΔgoxΔsfc* | *ΔgoxΔsfc* | This study |
| *ΔgoxΔicl1* | *ΔgoxΔicl1* | This study |
| *ΔgoxΔicl2* | *ΔgoxΔicl2* | This study |
| *ΔgoxΔasl* | *ΔgoxΔasl* | This study |
| *ΔgoxΔcps1* | *ΔgoxΔcps1* | This study |
| *ΔgoxΔcrz1* | *ΔgoxΔcrz1* | This study |
| ASL-H | *ΔgoxΔasl + ASL* | This study |
| CPS1-H | *ΔgoxΔcps1 + CPS1* | This study |
| CPS2L-H | *ΔgoxΔcps2l + CPS2L* | This study |
| CPS2S-H | *ΔgoxΔcps2s + CPS2S* | This study |
| CRZ1-H | *ΔgoxΔcrz1 + CRZ1-GFP* | This study |
| SFC-H | *ΔgoxΔsfc + SFC* | This study |
| e-PYC | *Δgox Δpks1 + PYC* | This study |
