# Supplementary material for: The ornithine-urea cycle involves fumaric acid biosynthesis in Aureobasidium pullulans var. aubasidani, a green and eco-friendly process for fumaric acid production
Source: Synth Syst Biotechnol. 2022 Oct 19;8(1):33–45. doi: 10.1016/j.synbio.2022.10.004 (PMC9647333; doi:10.1016/j.synbio.2022.10.004)
Supplement: Multimedia component 10 [file mmc10.doc]

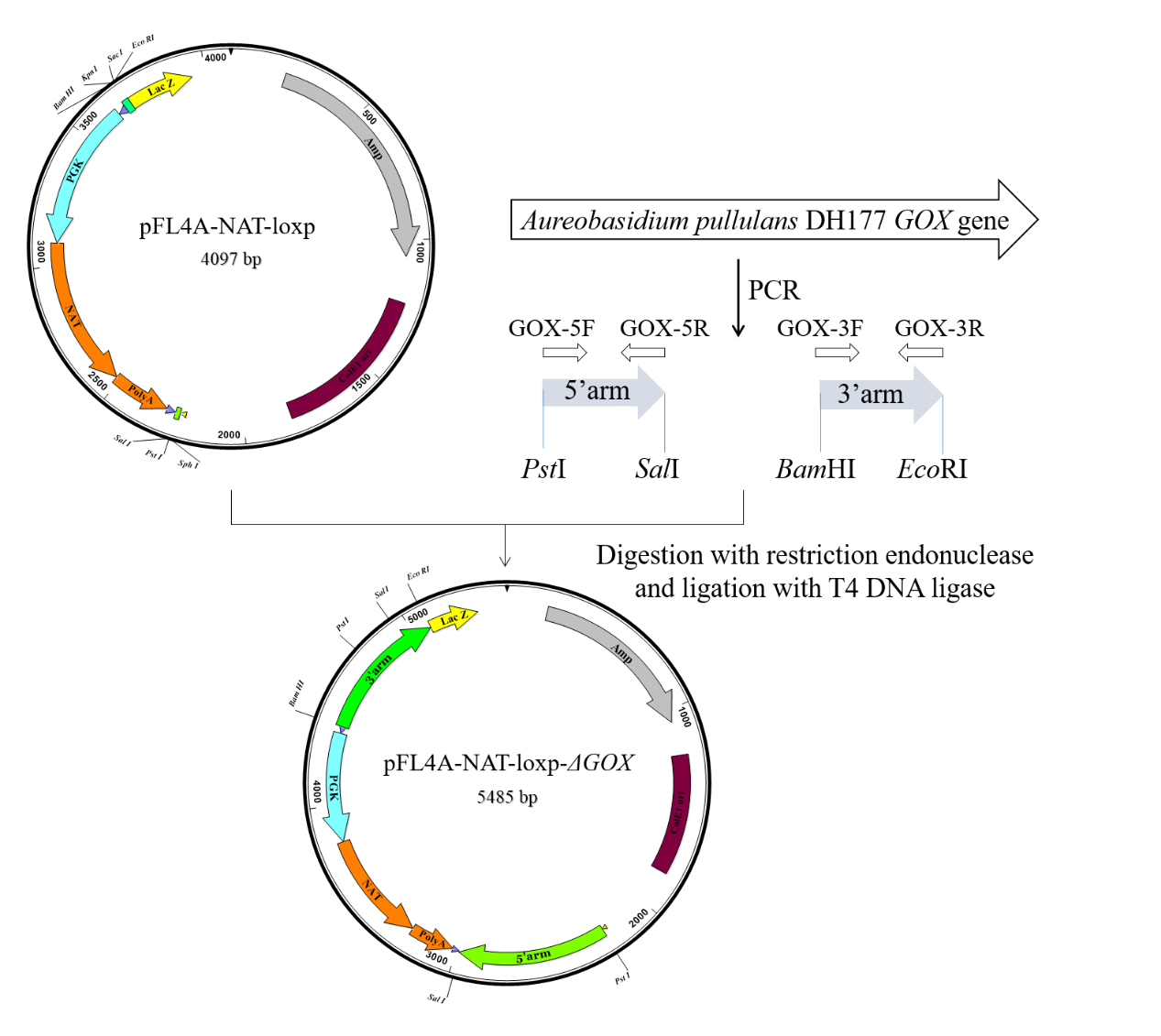


A


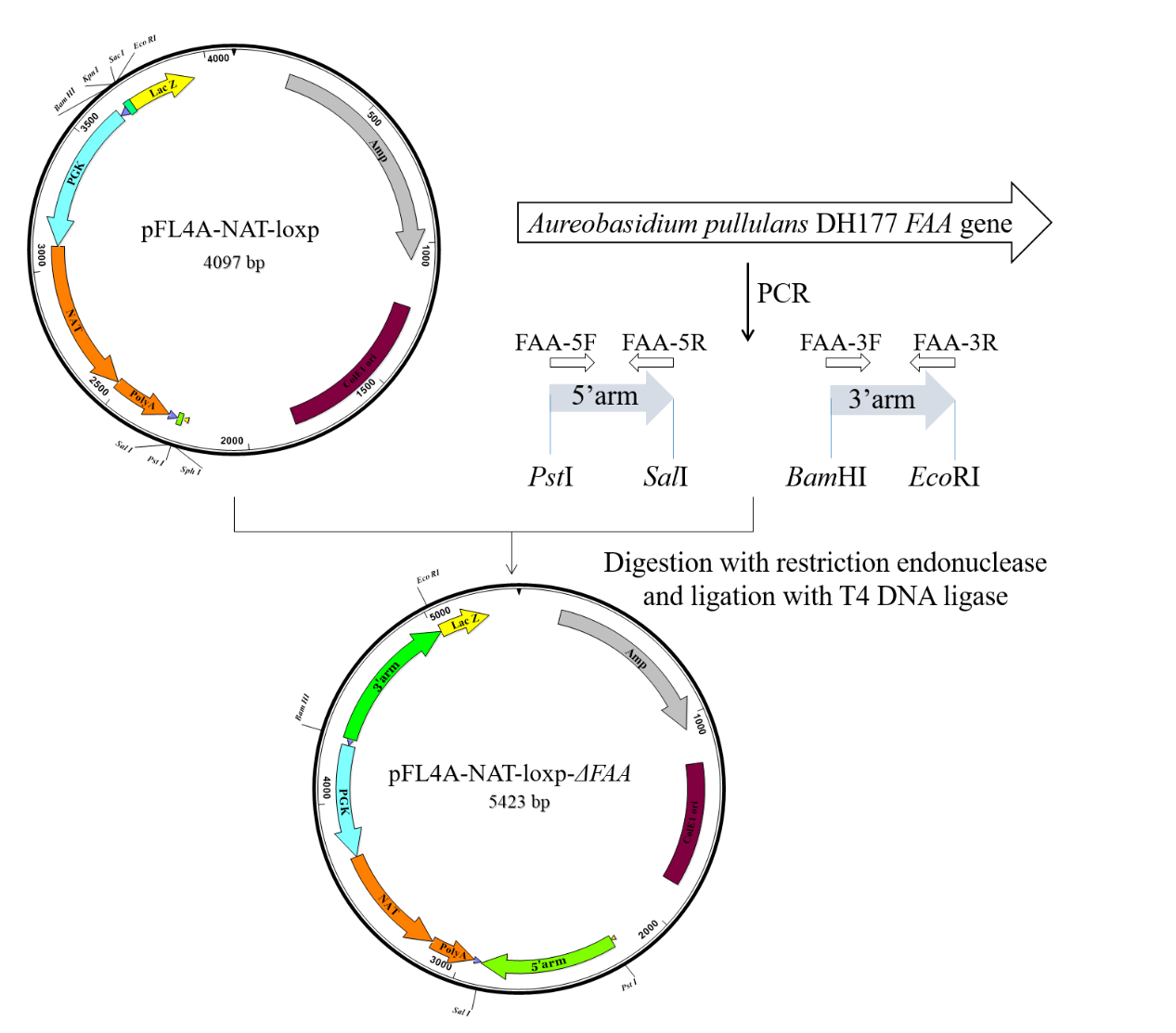


B


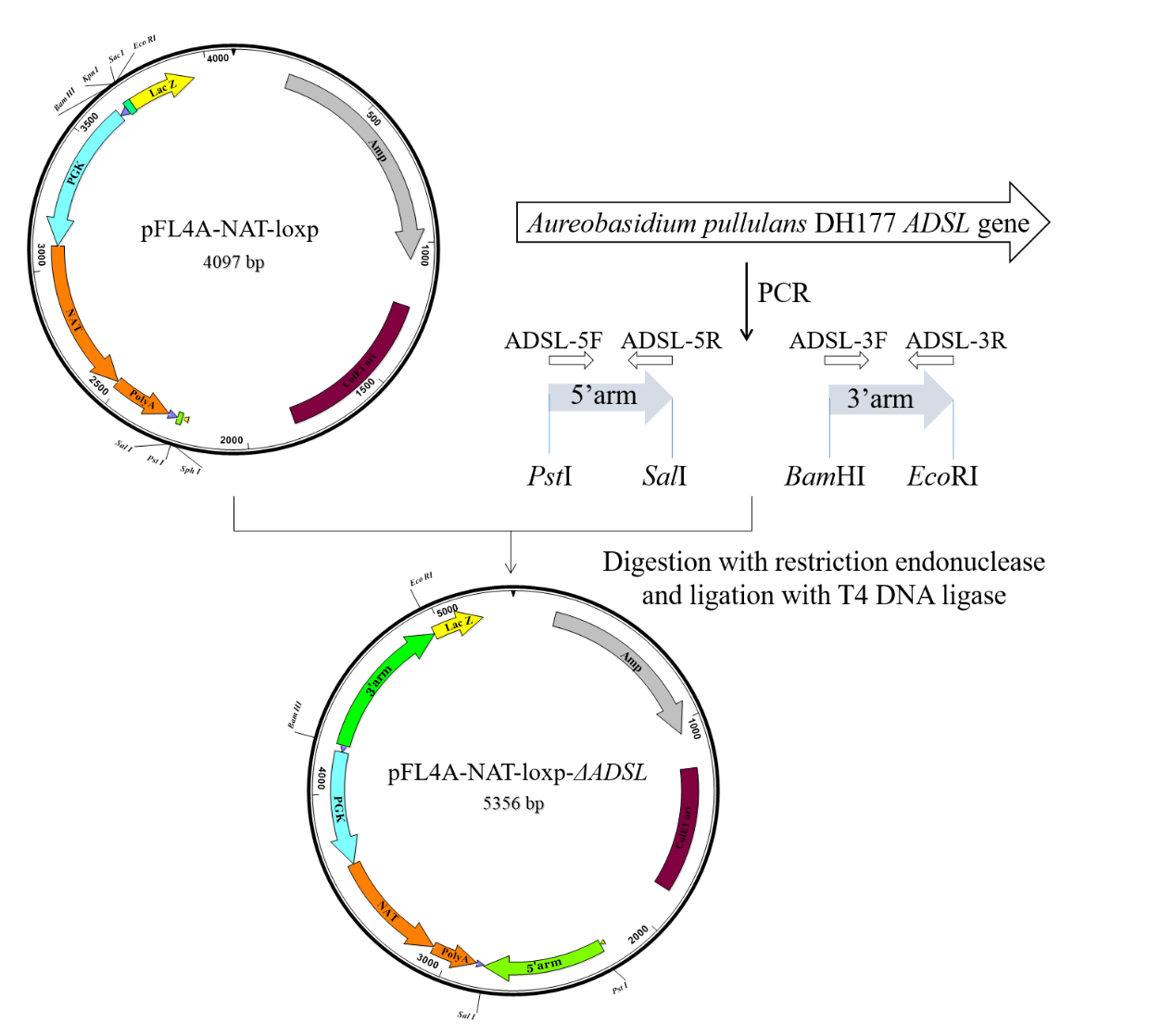


C


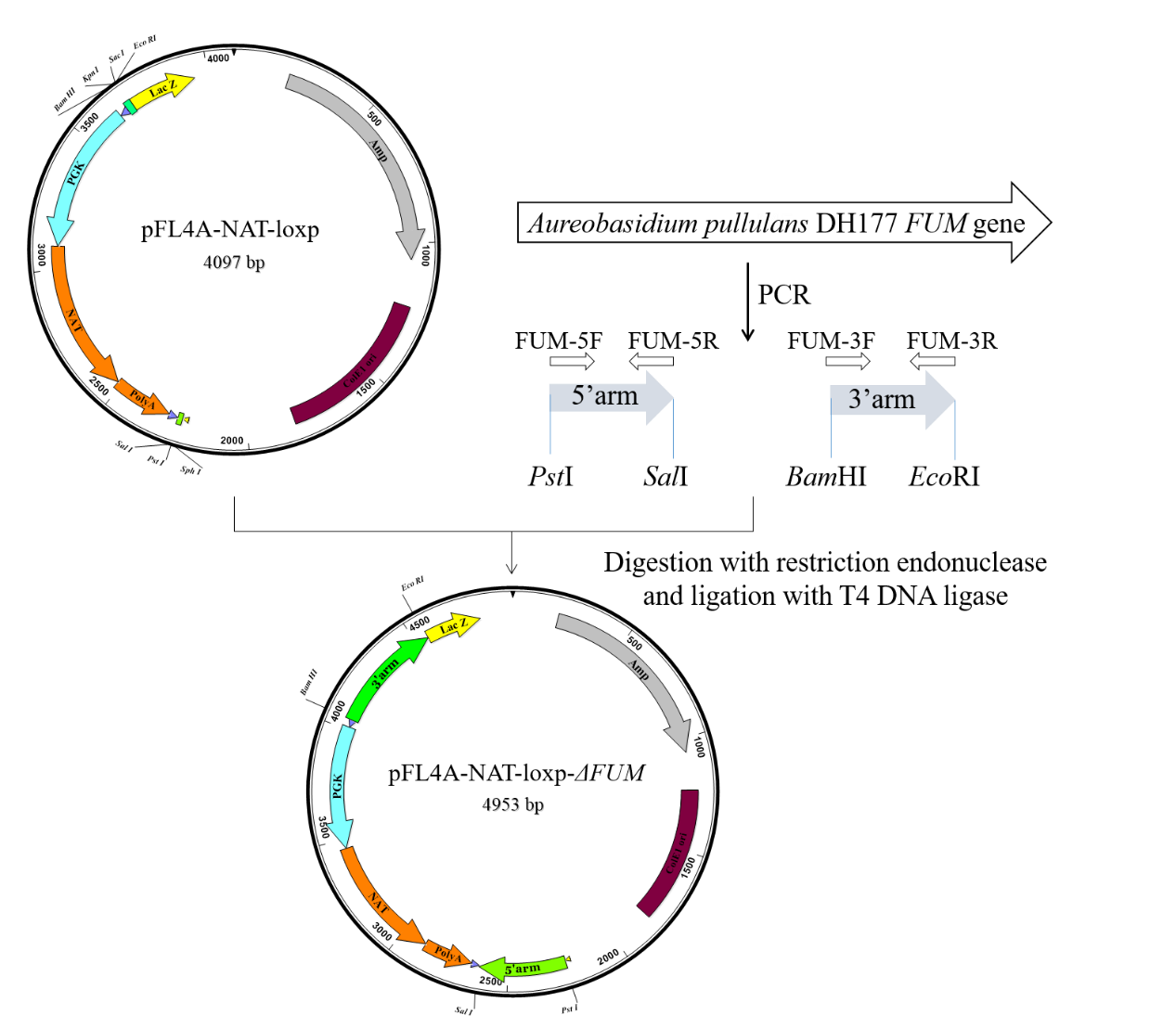


D


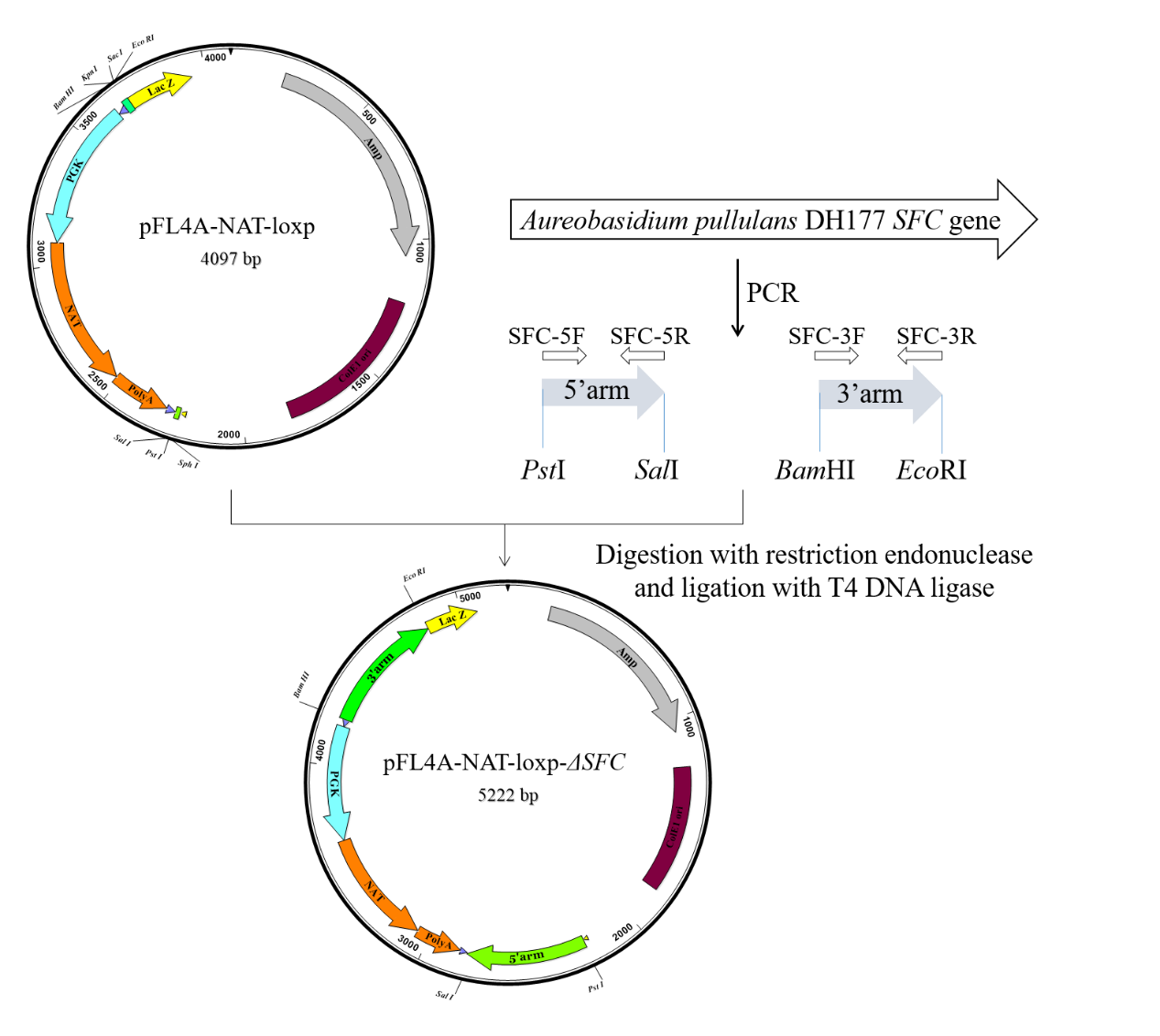


E


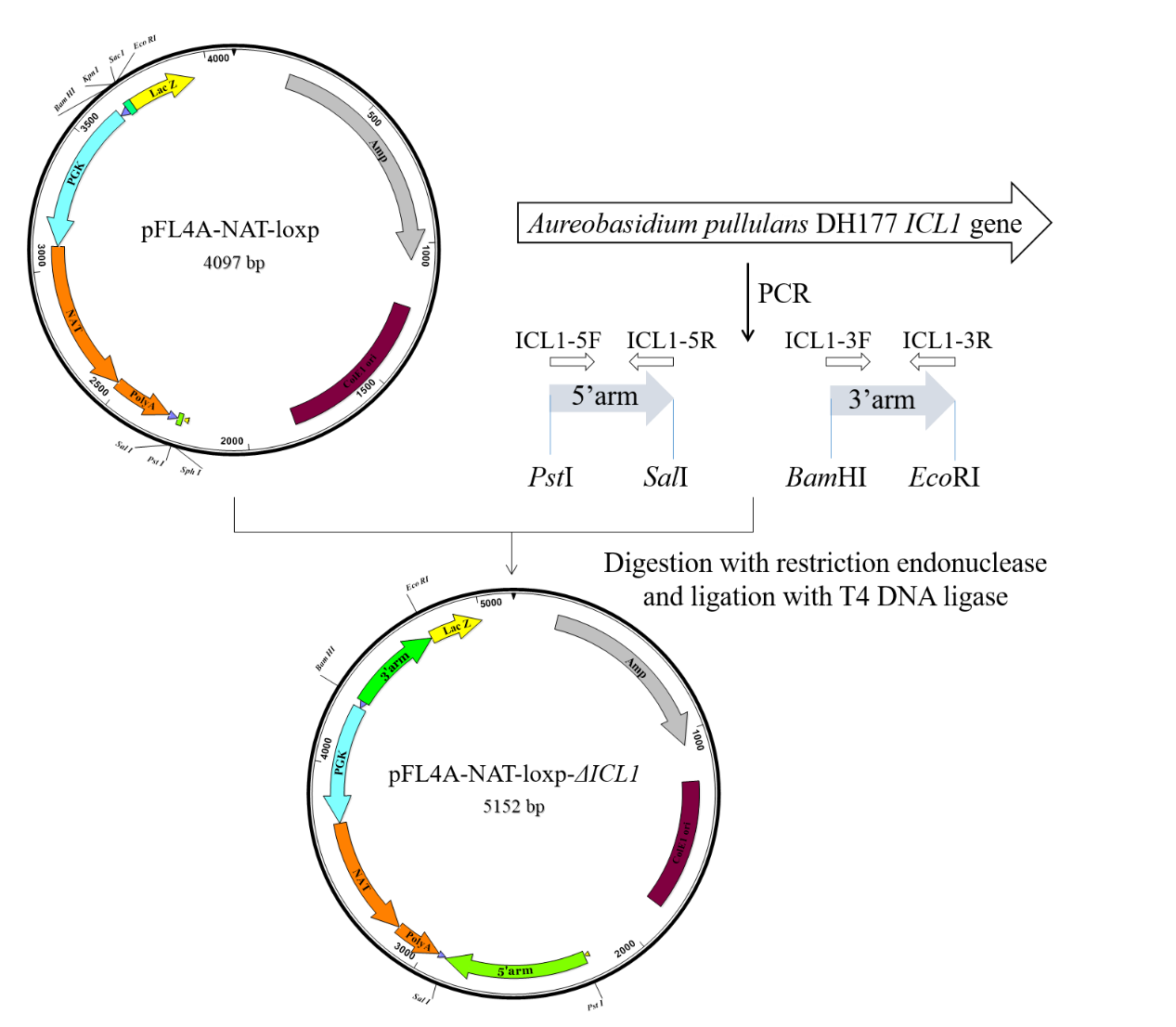


F


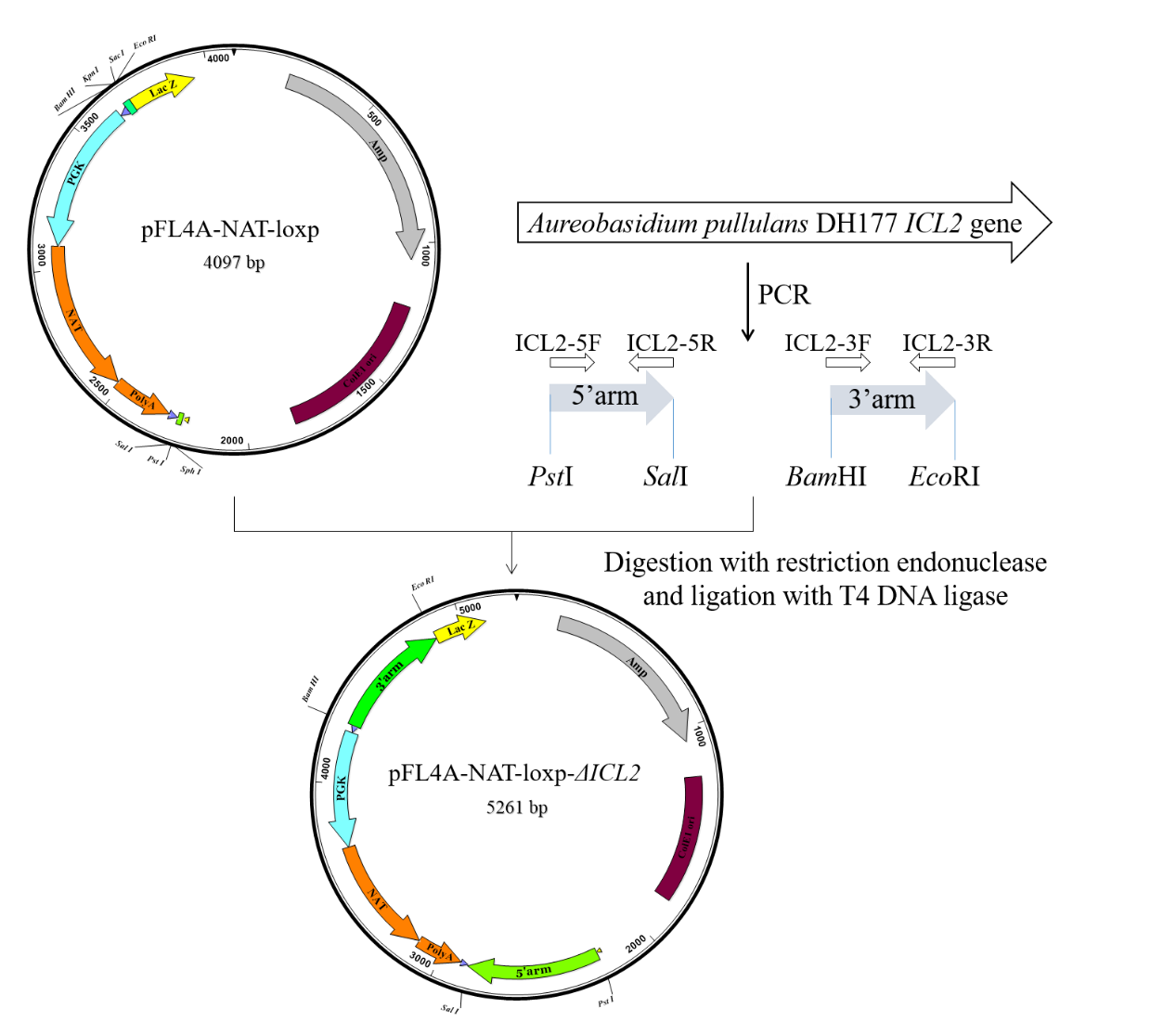


G


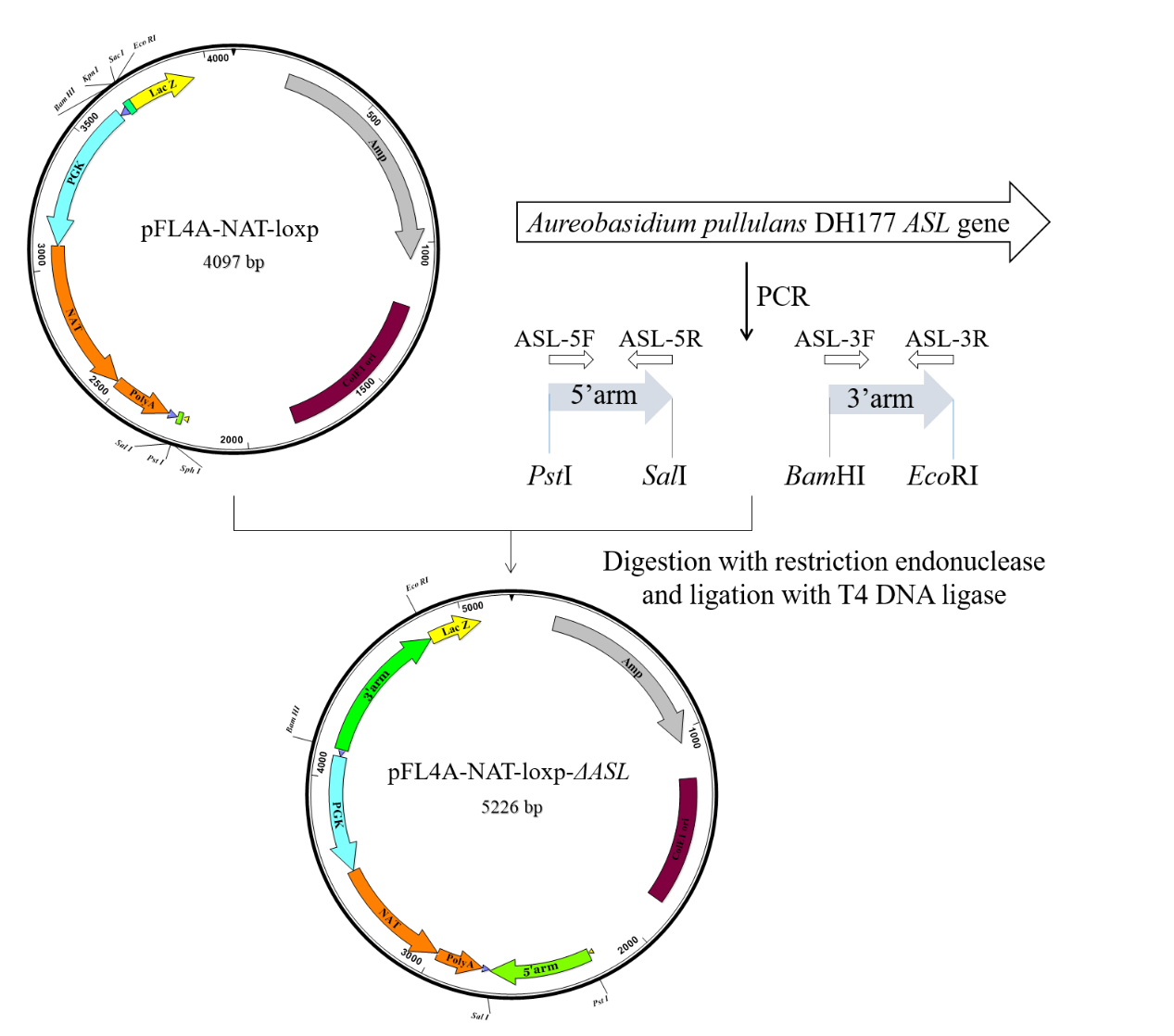


H


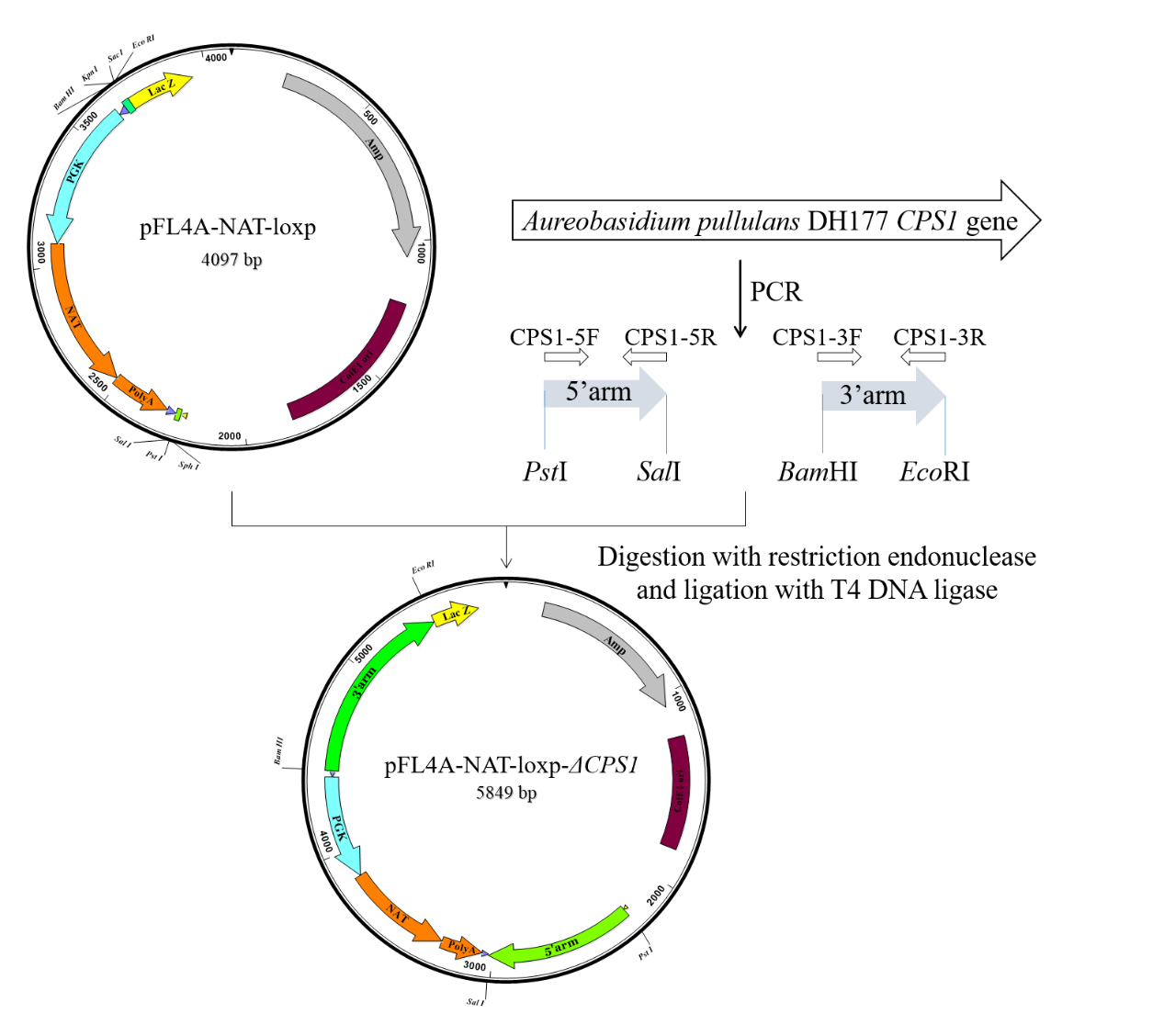


I


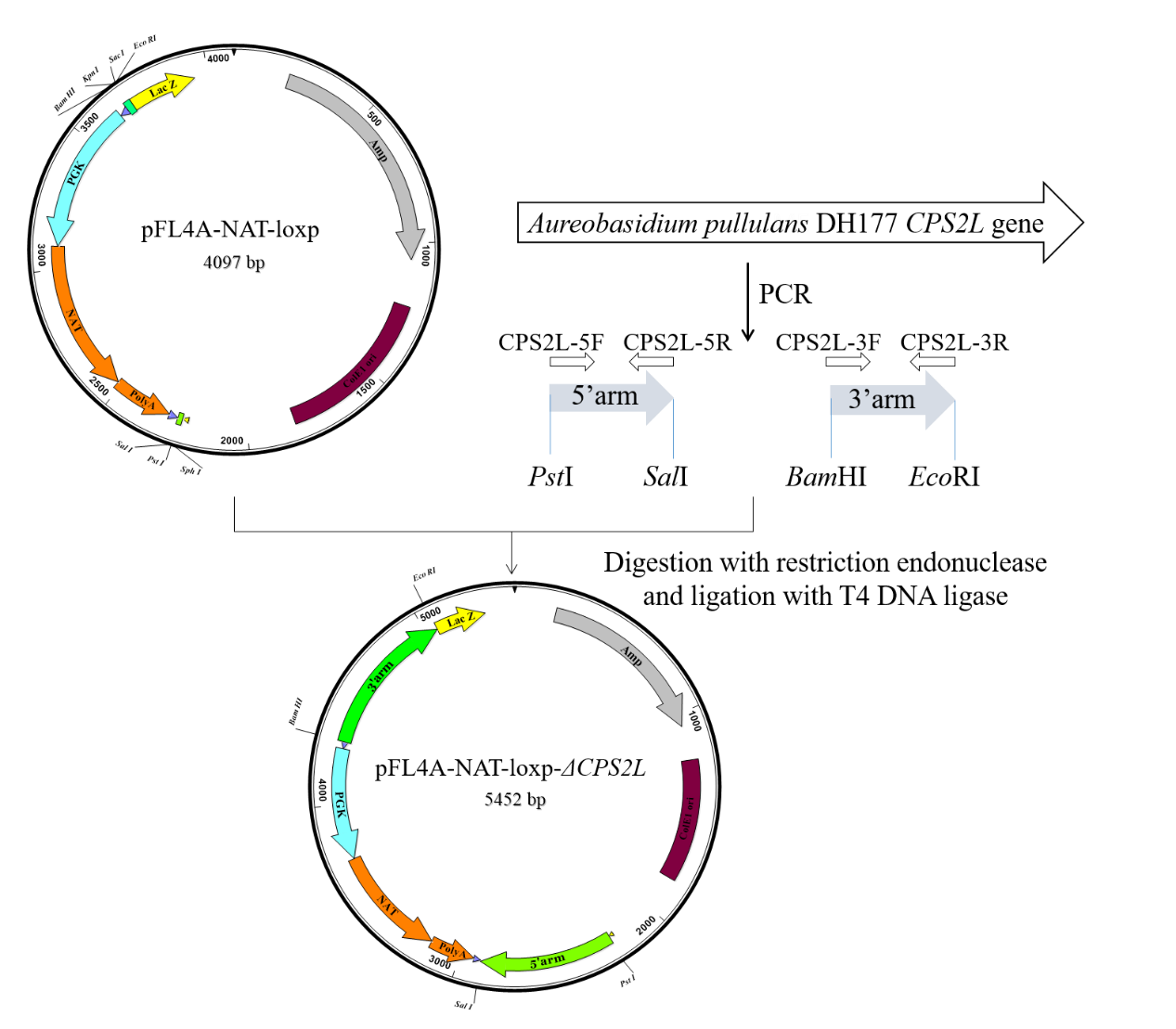


J


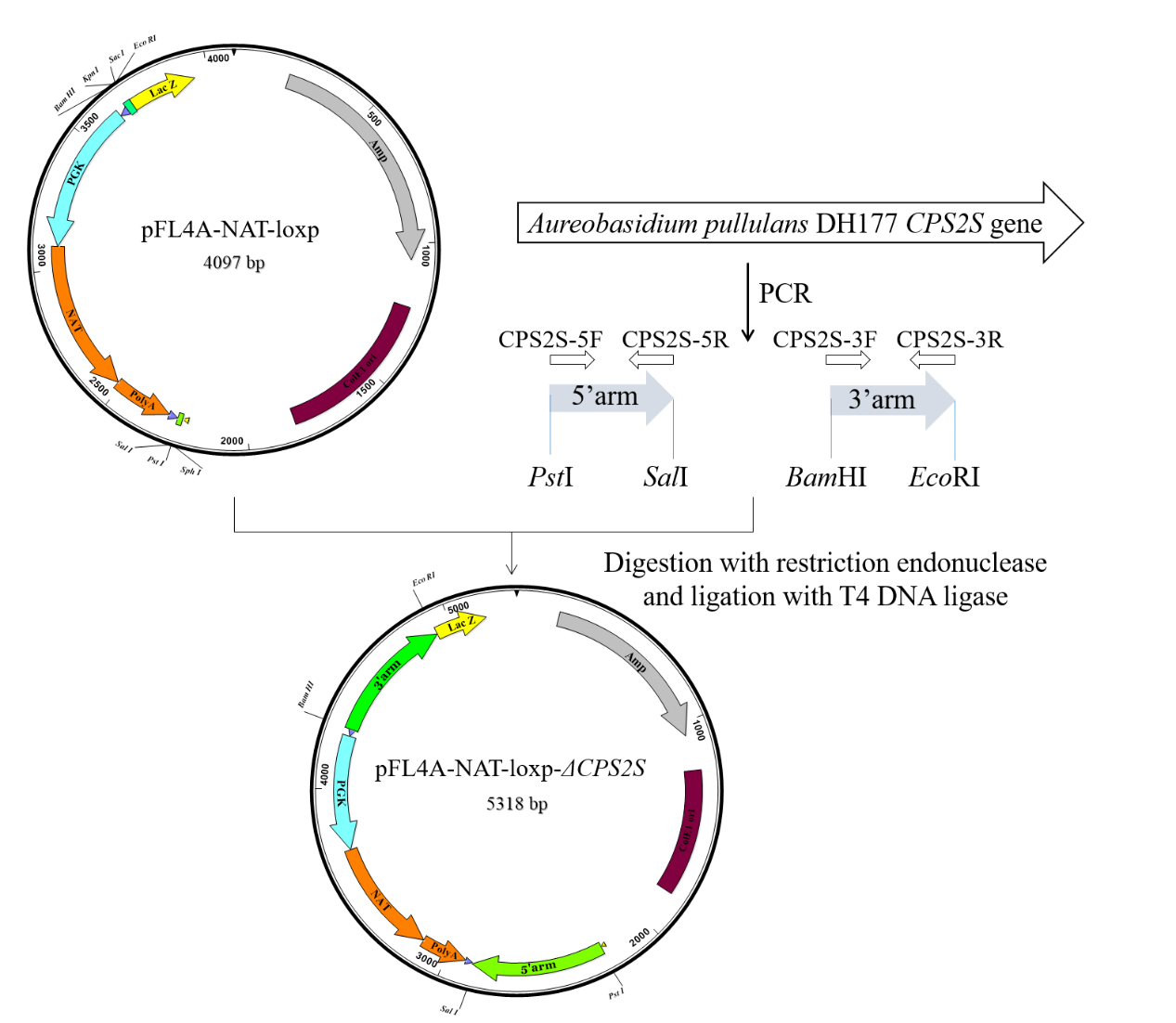


K


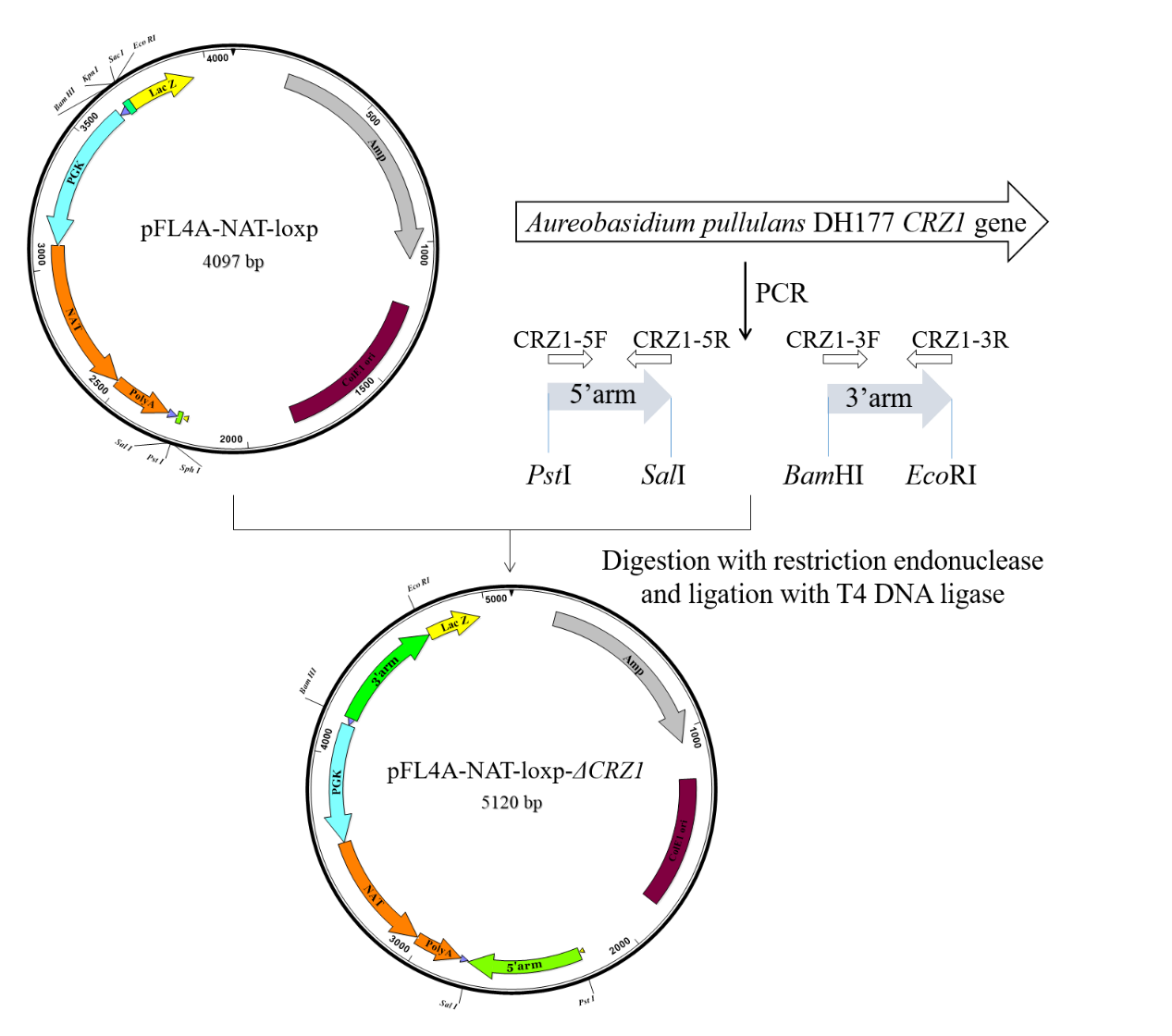


L


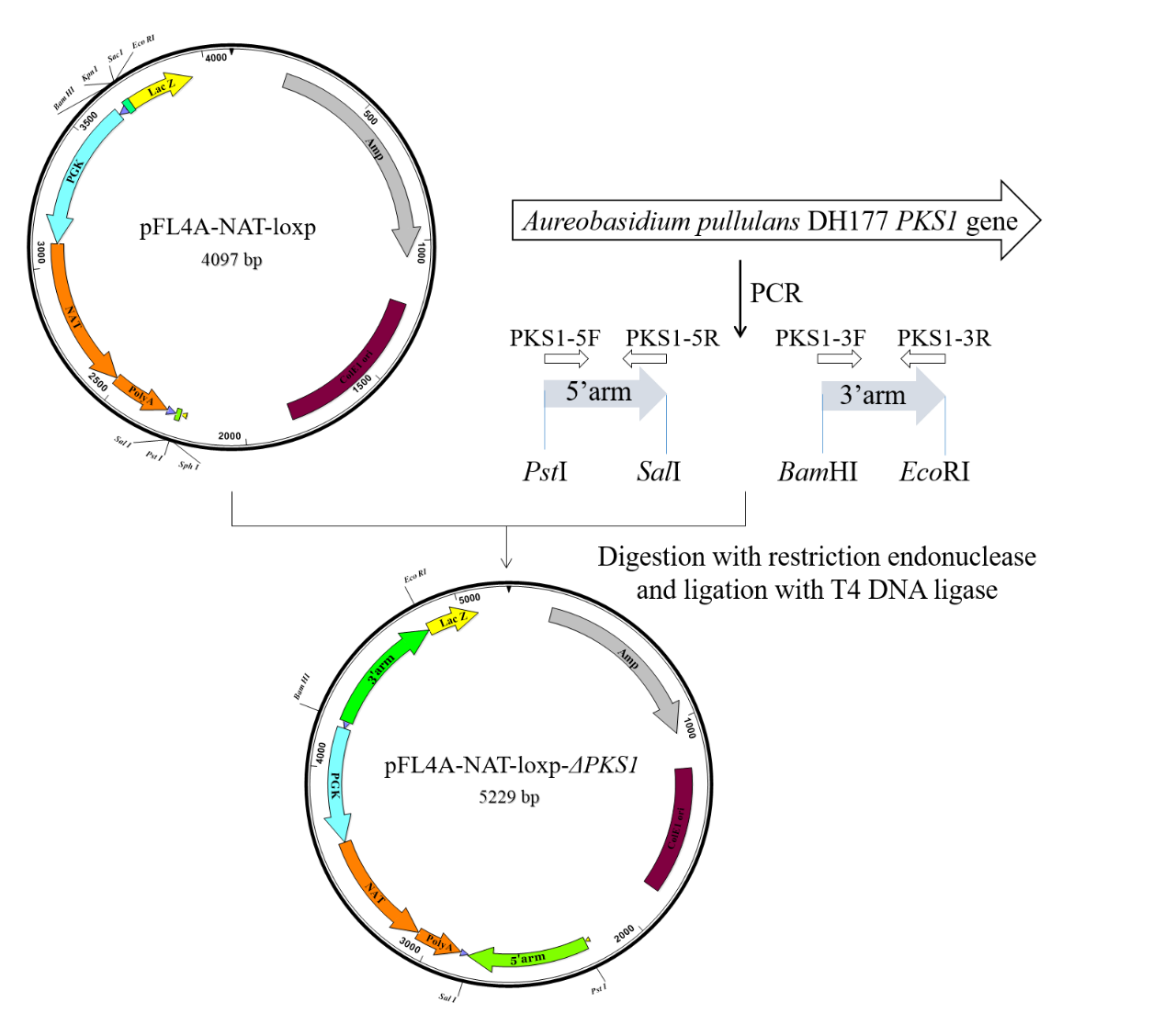


M


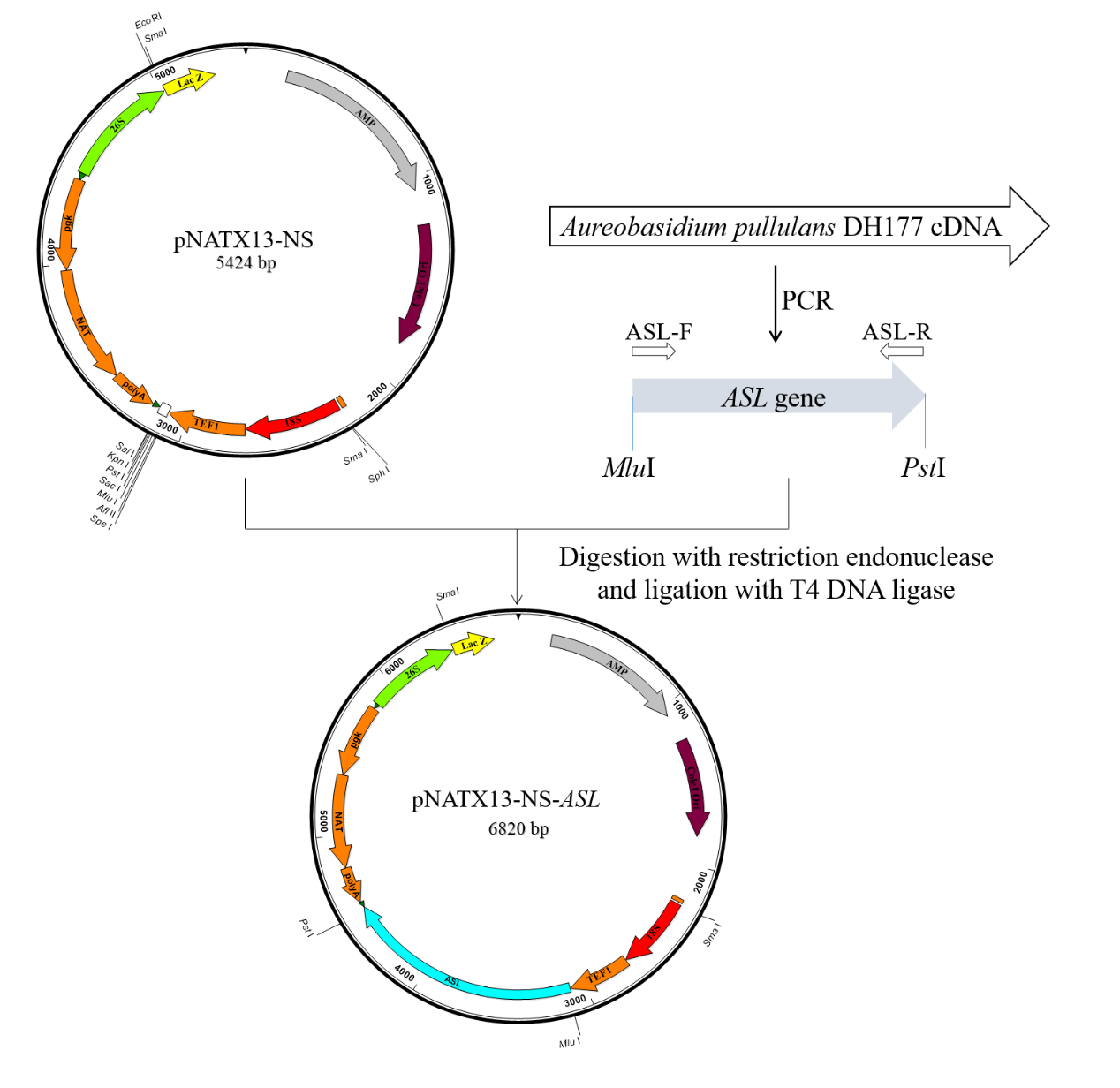


N


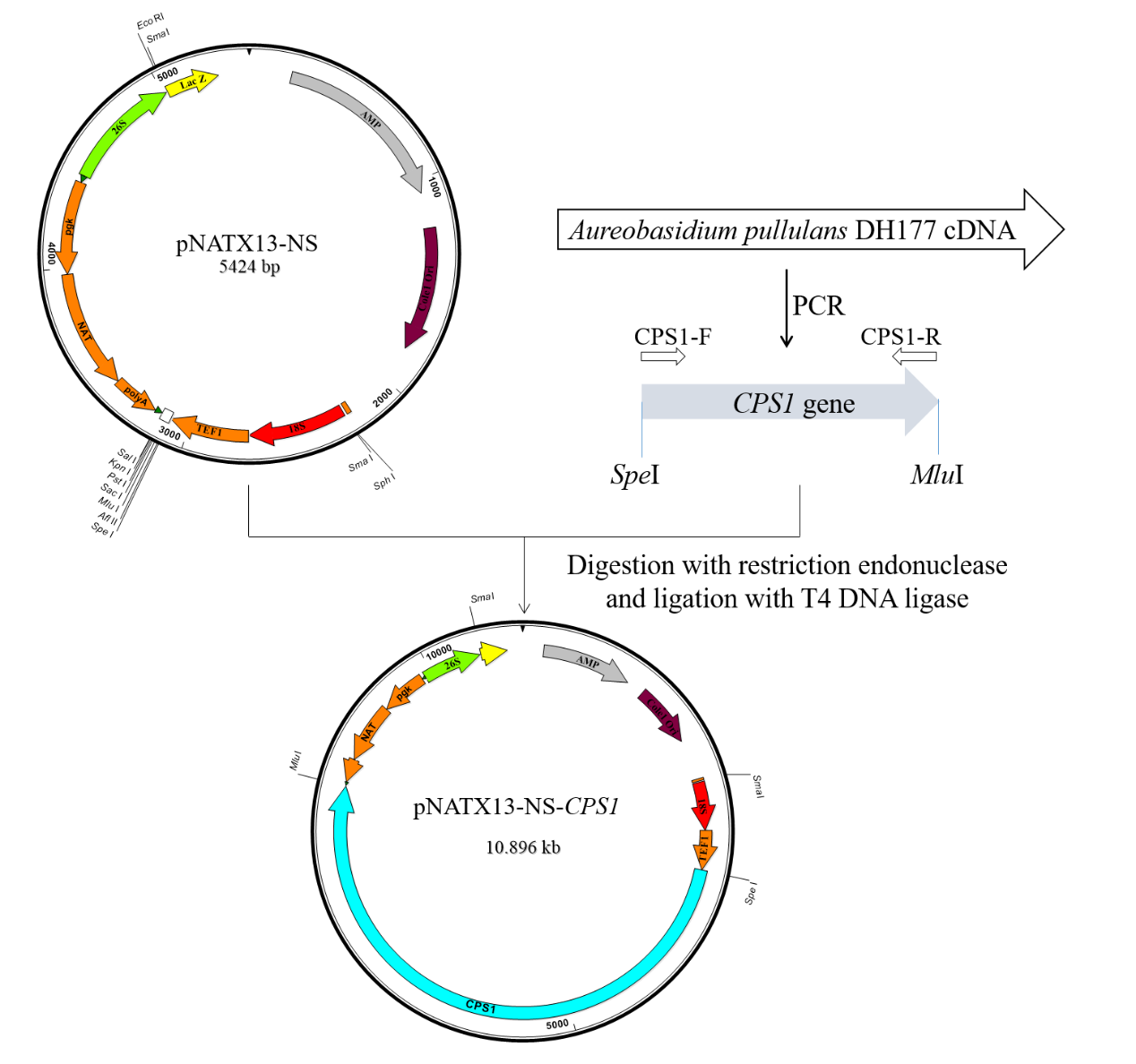


O


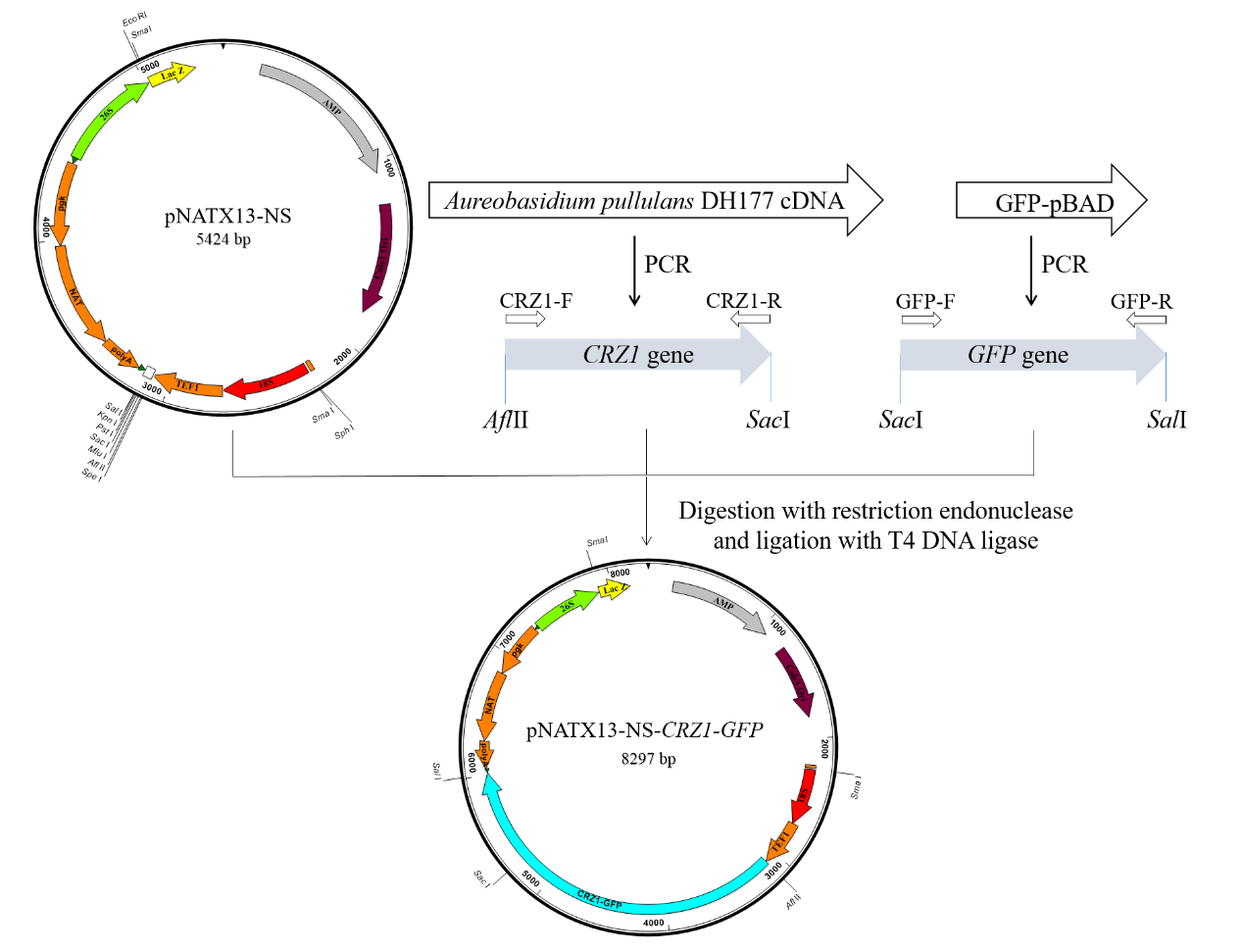


P


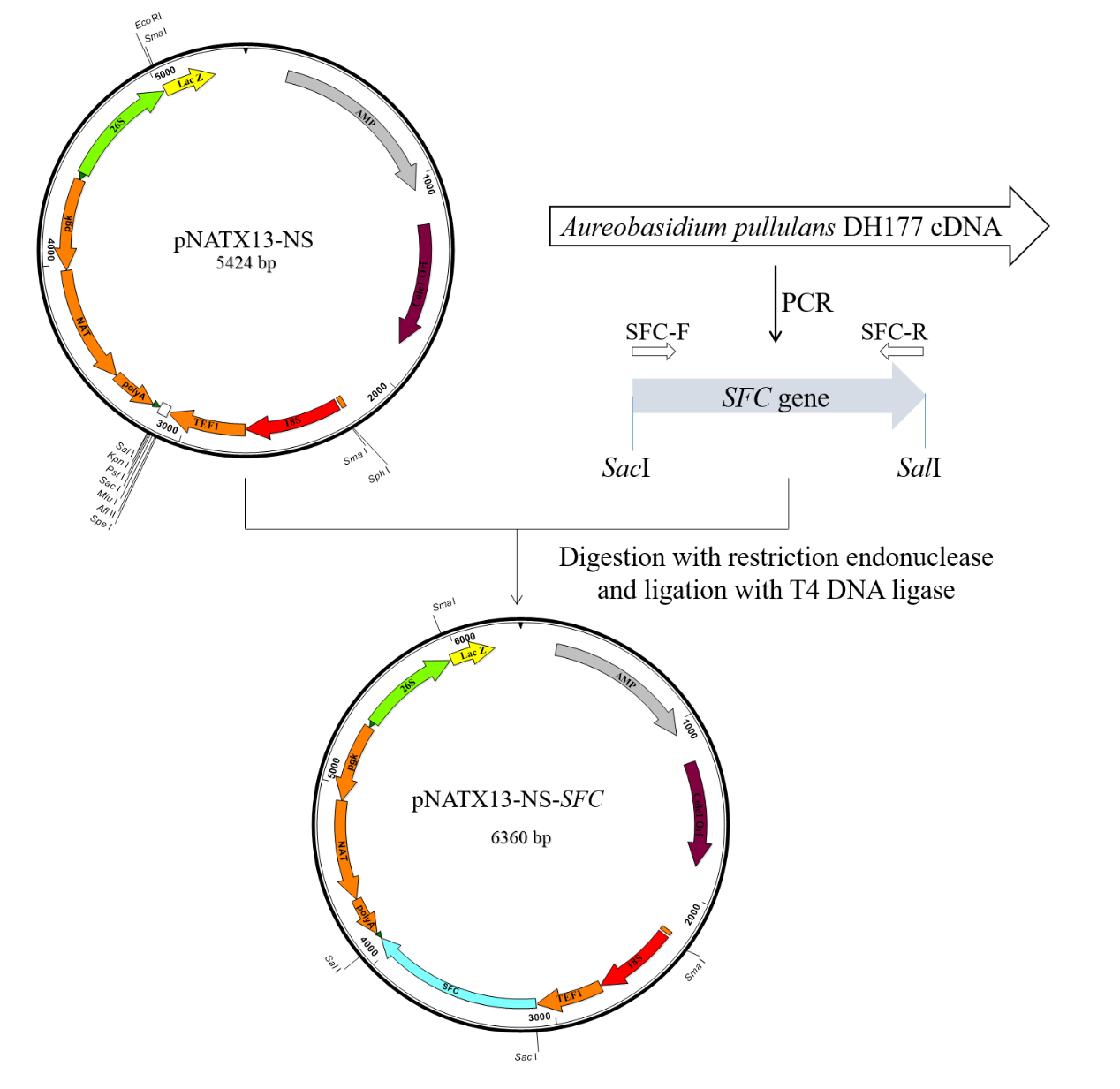


Q


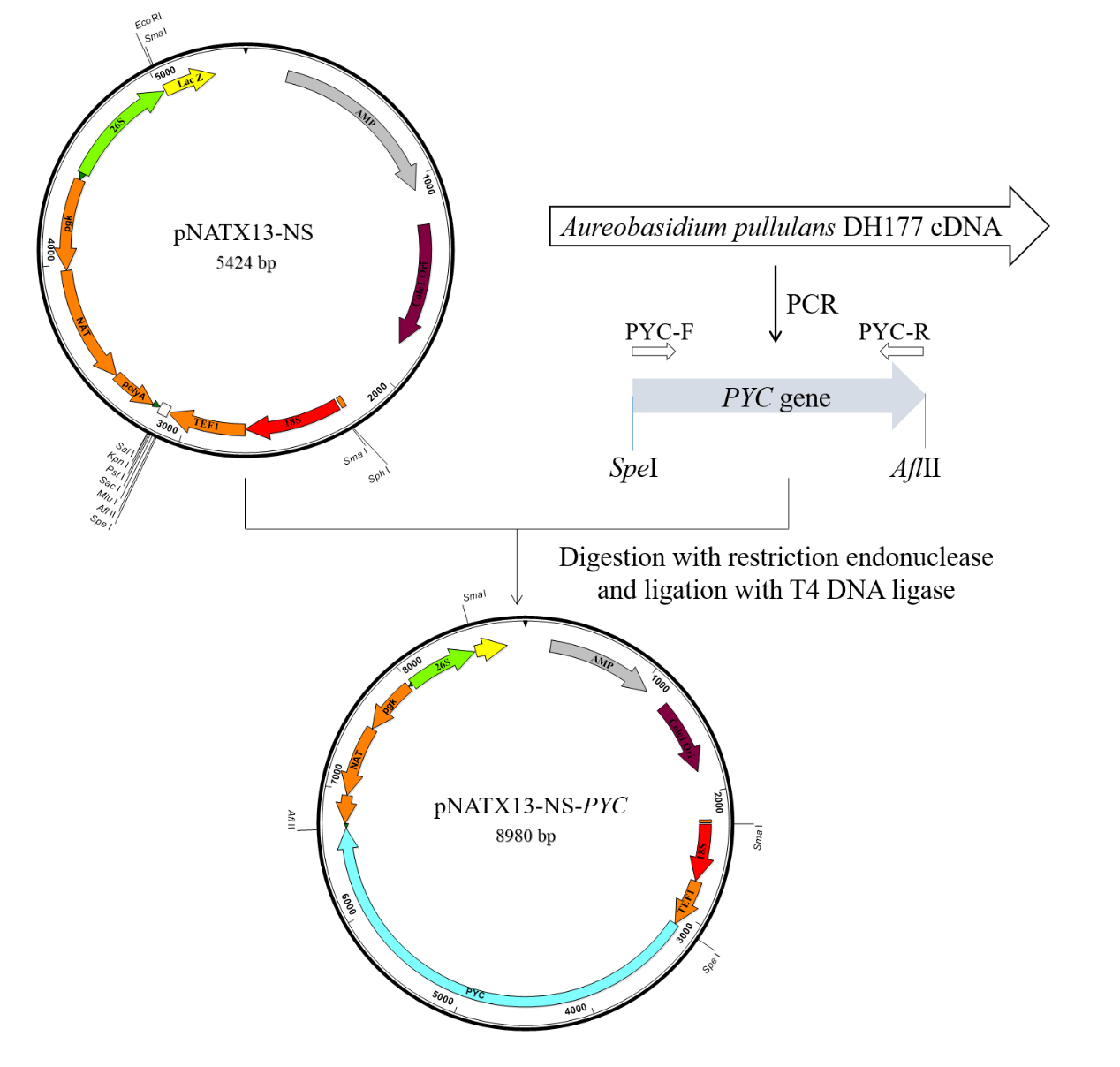


R


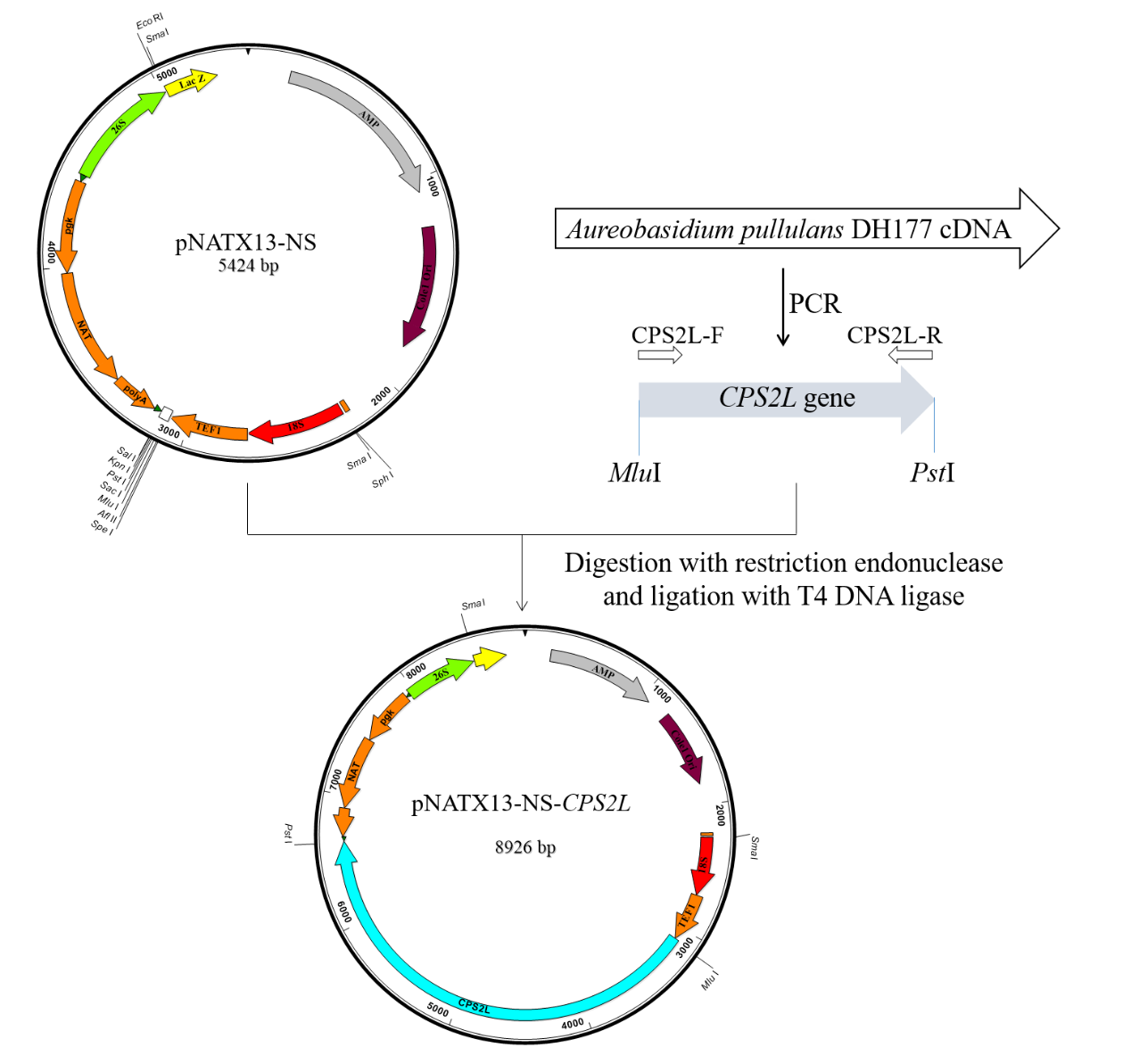


S


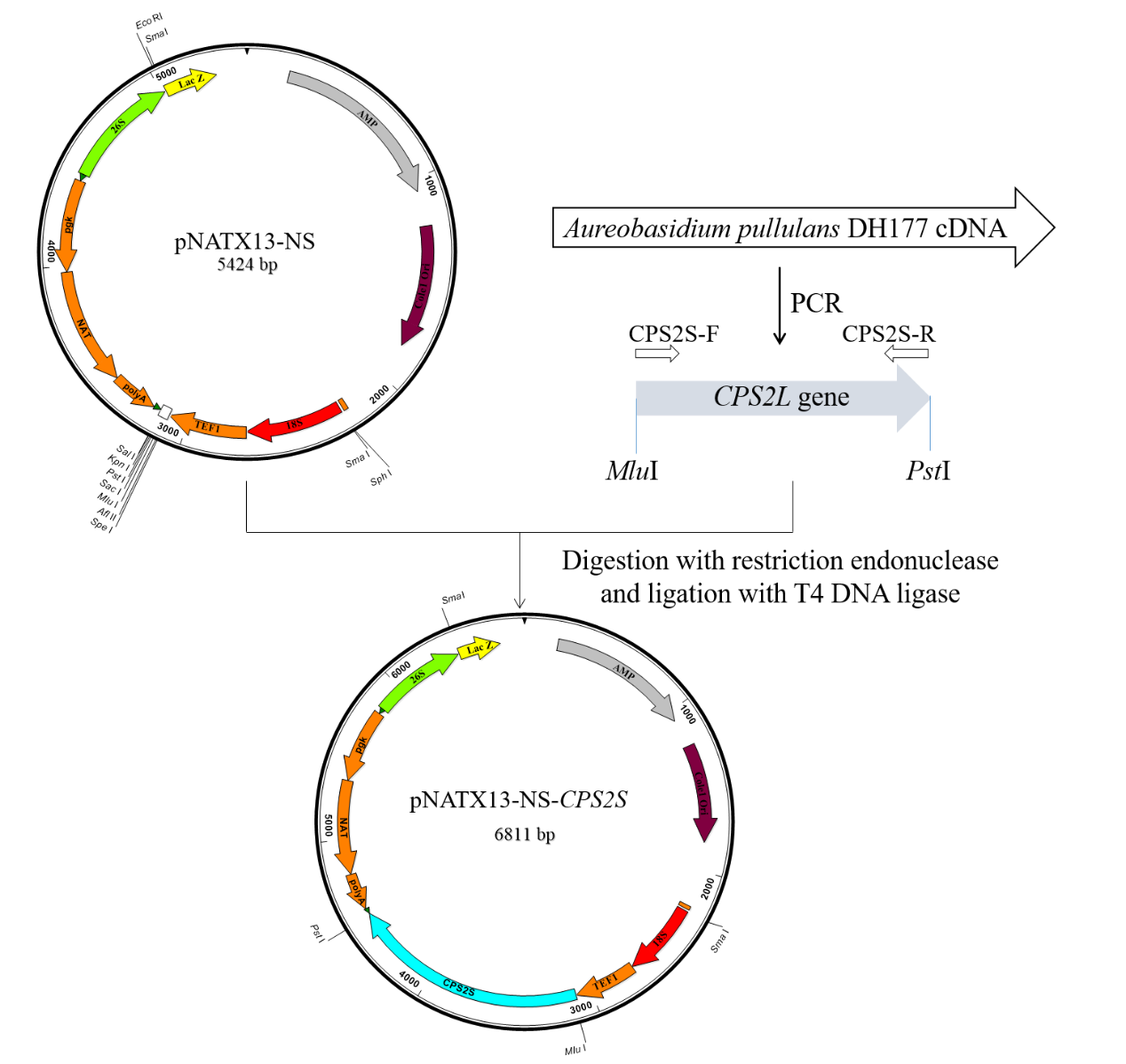


T

**Fig. S1** Construction of the knock-out vectors pFL4A-NAT-loxp-*△GOX* (A), pFL4A-NAT-loxp-*△FAA* (B), pFL4A-NAT-loxp-*△ADSL* (C), pFL4A-NAT-loxp-*△FUM* (D), pFL4A-NAT-loxp-*△SFC* (E), pFL4A-NAT-loxp-*△ICL1* (F), pFL4A-NAT-loxp-*△ICL2* (G), pFL4A-NAT-loxp-*△ASL* (H), pFL4A-NAT-loxp-*△CPS1* (I), pFL4A-NAT-loxp-*△CPS2L* (J), pFL4A-NAT-loxp-*△CPS2S* (K), pFL4A-NAT-loxp-*△CRZ1* (L), pFL4A-NAT-loxp-*△PKS1* (M) and the knock-in vectors pNATX13-NS-*ASL* (N), pNATX13-NS-*CPS1* (O), pNATX13-NS-*CRZ1-GFP* (P), pNATX13-NS-*SFC* (Q), pNATX13-NS-*PYC* (R), pNATX13-NS-*CPS2L* (S), pNATX13-NS-*CPS2S* (T).
