# Supplementary material for: The ornithine-urea cycle involves fumaric acid biosynthesis in Aureobasidium pullulans var. aubasidani, a green and eco-friendly process for fumaric acid production
Source: Synth Syst Biotechnol. 2022 Oct 19;8(1):33–45. doi: 10.1016/j.synbio.2022.10.004 (PMC9647333; doi:10.1016/j.synbio.2022.10.004)
Supplement: Multimedia component 11 [file mmc11.doc]

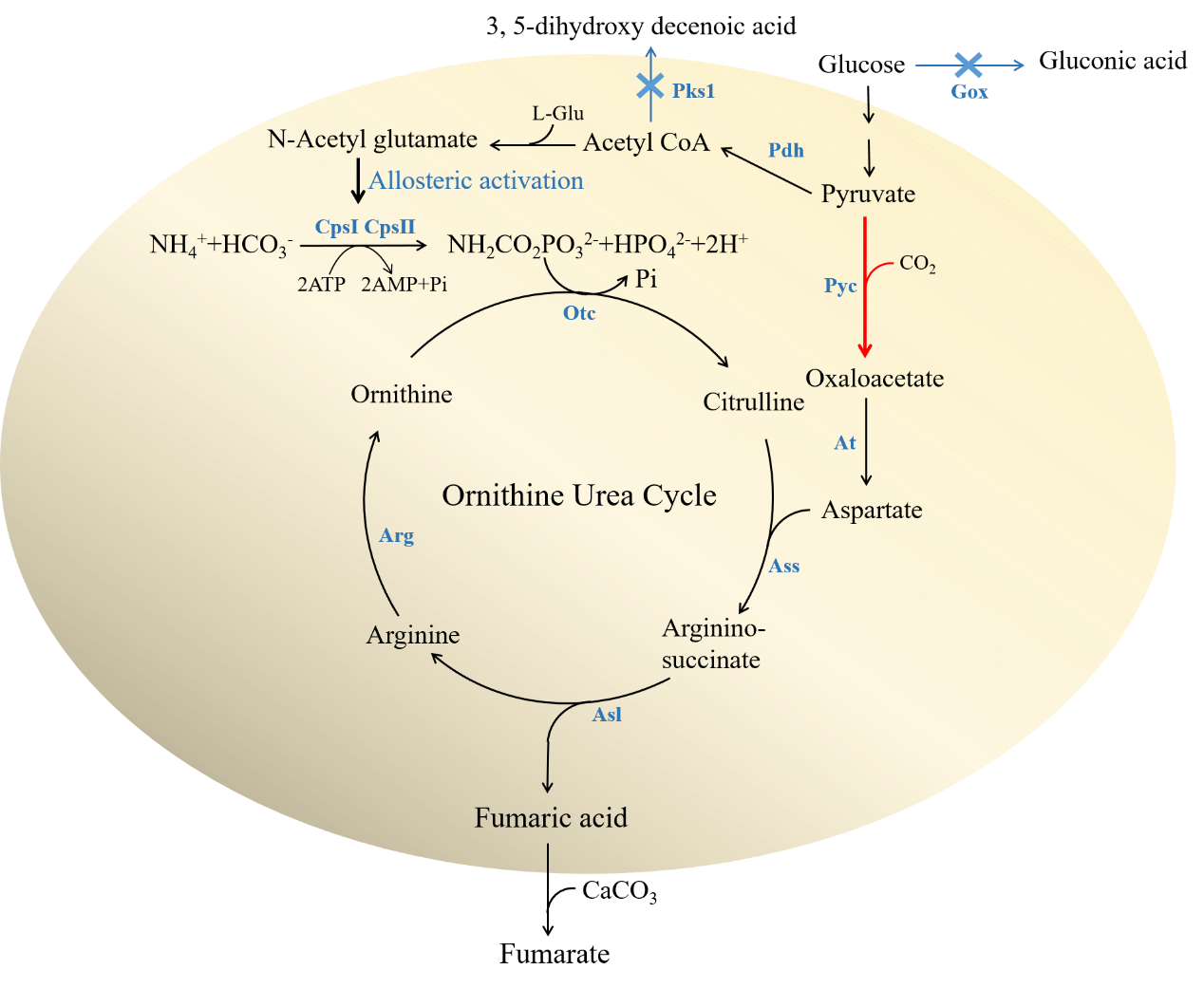


**Fig. S2** The genetical strategies for enhanced fumarate production. × means deletion. Pks1, polyketide synthase; Gox, glucose oxidase; Pdh, pyruvate dehydrogenase; any other enzymes have been described in Fig. 1.
