# Supplementary material for: The ornithine-urea cycle involves fumaric acid biosynthesis in Aureobasidium pullulans var. aubasidani, a green and eco-friendly process for fumaric acid production
Source: Synth Syst Biotechnol. 2022 Oct 19;8(1):33–45. doi: 10.1016/j.synbio.2022.10.004 (PMC9647333; doi:10.1016/j.synbio.2022.10.004)
Supplement: Multimedia component 12 [file mmc12.docx]

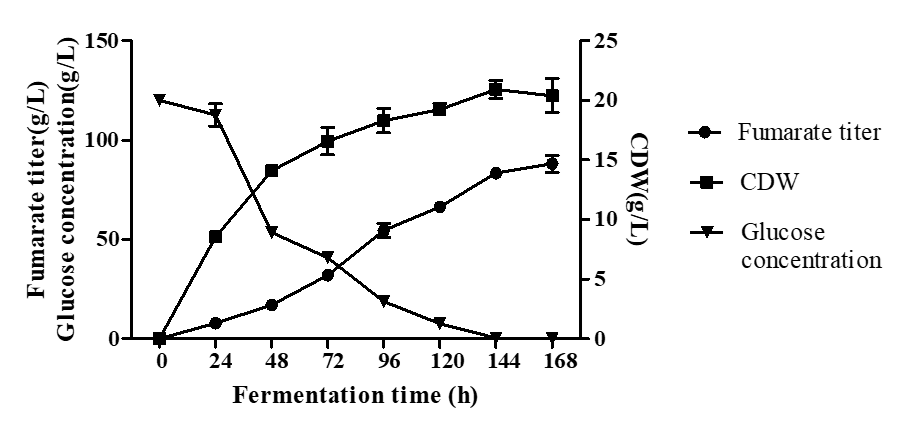


**Fig. S1** Time course of the changes in fumarate titer, cell growth of the strain e-PYC and residual glucose concentration during the cultivation at flask level. Data are given as mean ± SD, n=3.
